# Synthesis of copper schiff base nanocomplex as green inhibitor for 316 L stainless steel corrosion in an aggressive acidic environment

**DOI:** 10.1038/s41598-025-21933-3

**Published:** 2025-10-20

**Authors:** Ghalia A. Gaber, Shimaa Hosny, Fakiha El-Taib Heakal

**Affiliations:** 1 Chemistry Department, Faculty of Science (Girls), Al-Azhar University, Cairo11754, Nasr City, Egypt; 2 Chemistry Department, Faculty of Science, New Valley University, El-Kharga, 72511 Egypt; 3 Chemistry Department, Faculty of Science, Cairo University, Giza, 12613 Egypt

**Keywords:** Green inhibitor, Cu nano-complex, 316L stainless steel, Electrochemical techniques, DFT calculation, MD simulations, Chemistry, Materials science, Nanoscience and technology

## Abstract

Using inhibitors is a typical way to mitigate metal degradation by forming barriers or altering reactions. Herein, the nanocomplex of copper(II) ion with 1-((E)-(2-mercaptophenylimino) methyl) naphthalen-2-ol (H_2_L) Schiff base ligand was successfully synthesized via an environmentally friendly approach using *Coriandrum Sativum* (CS) extract in ethanol. According to transmission electron microscopy (TEM), the average particle size of the produced nanocomplex was 12.71 nm. Using various electrochemical and physical techniques, namely, open-circuit potential (OCP), impedance spectroscopy (EIS), potentiodynamic polarization (PDP), and SEM/EDX methods, the inhibitory effect of green Cu nanocomplex (Cu_2_L_2_) on the corrosion of 316 L stainless steel in a harsh mixed acid solution (1 M HCl + 1 M HNO_3_) was scrutinized. The findings revealed that the Cu nanocomplex exhibited mixed-type inhibition behavior, with a maximum efficiency of 85.56% achieved at 600 ppm addition. EIS results indicated an increase in the charge transfer resistance and a decrease in the double-layer capacitance by increasing Cu_2_L_2_ concentration in the ambient aggressive acidic solution. Isotherm fitting analysis suggested that Cu nanocomplex adsorption on the stainless steel surface followed the Langmuir isotherm model. Density functional theory (DFT) calculations further supported the experimentally obtained findings. The molecular dynamics (MD) simulations also endorsed that the Cu_2_L_2_ inhibitor lay flat on the metal surface, indicating better surface coverage.

**Supplementary Information:**

The online version contains supplementary material available at 10.1038/s41598-025-21933-3.

## Introduction

Nanostructured materials have emerged as promising corrosion inhibitors due to their distinctive structural and functional features. Among those materials are the nano-complexes characterized by their large surface area, tunable organic ligands, and redox-active centers. These render them adsorbed onto metal surfaces, produce protective coatings, and inhibit electrochemical corrosion processes^1,2^. Numerous studies have successfully proven the application of nanomaterials as corrosion inhibitors^3,4^. Recent research showed that metal and oxide nanoparticles can improve the corrosion resistance of metallic materials. Asafa et al.^5^ have evaluated the effect of silver nanoparticles (AgNP) on the suppression of mild steel, stainless steel, and aluminum corrosion in 1 M HCl. Adding AgNP solution increased the inhibition efficiency by 52% for mild steel, 70% for stainless steel, and 62% for aluminum. Singh et al.^6^ have reviewed the role of silica nanoparticles in improving the mechanical and anticorrosive properties of coatings, highlighting their role in pore filling and adhesion enhancement.

Stainless steel alloys are widely employed in various industrial applications, including chemical production, food processing, and oil refining^7^. Corrosion in stainless steel happens when the protective oxide layer on the surface deteriorates, which is frequently caused by environmental conditions such as chloride ions, high temperatures, or mechanical damage. Those effects are usually conducive to localized corrosion, such as pitting and crevice corrosion, especially in chloride-rich environments^8^.

Hydrochloric and nitric acids are corrosive solutions widely employed in industrial procedures such as acid cleaning, acid descaling, steel pickling, boiler cleaning, oil well oxidization, ion exchange recovery, and waste treatment. Using an HCl + HNO₃ solution replicates very corrosive settings. Hot-rolled metals in steel manufacture produce oxide scales (e.g., Fe₃O₄, Fe₂O₃) that need to be removed through pickling. While HCl is often used for pickling carbon steel, stainless steel, and high-alloy steels usually demand stronger or mixed HNO₃. Residual HCl in process streams can create aggressive mixed-acid conditions, accelerating corrosion. These conditions are typical of industrial settings where stainless steel components are frequently subjected to oxidizing and chloride-rich environments^9–12^. Aqua regia offers both oxidizing (by HNO₃) and chloride-rich (via HCl) conditions, making it a helpful tool for studying pitting and corrosion in stainless steel. These conditions are challenging for protective coatings, making them an ideal test for assessing the efficacy of our nano Schiff-based protective layer.

More specifically, electrochemical corrosion of steel occurs when the metallic surface releases ions into its ambient environment, such as a layer of moisture in the presence of oxygen^13^. In a wet air environment, iron in steel progressively changes into oxides, known as rust. Most metals and alloys are thermodynamically unstable as they acquire excess energy during metallurgical processing and purification^13,14^. Therefore, corrosion is a natural ability of metallic materials to form relatively more stable compounds such as oxides, hydroxides, sulfides, chlorides, etc. Metallic oxides are generally more energetically stable than metals themselves.

Because of its excellent corrosion resistance, stainless steel is extensively employed in several fields. Chloride ions are primarily responsible for the failure of stainless-steel equipment, particularly in cooling water systems^14–16^. In many industrial and natural settings, the chloride anion is the most often hostile ion^17^. The presence of this aggressive ion can cause the passive films on stainless steel objects to break down, thereby interrupting their passivity.

Many authors claimed that stainless steel’s exceptional resistance to corrosion in a variety of aqueous solutions stems from its capability to shield itself from corrosive environments, by forming a thin passive film that is composed of a mixture of iron and chromium oxides, with the chromium oxide enrichment located at the metal/film interface and the hydroxide, and water-containing compounds located at the passive films outermost region^18–22^. The composition of this film varies depending on the alloy type and temperature. This coating, which stabilizes all stainless steel, is thought to be nonporous, insoluble, passive, and self-healing in the event of an attack^22,23^. Stainless steel possesses solution potentials comparable to noble metals in passivity-friendly conditions^24^.

As a result, scientists and researchers are looking to reduce corrosion by adding anticorrosive chemicals. Organic molecules adsorb onto the stainless steel surface, forming a barrier layer that inhibits corrosion^25,26^. Creating nanoporous oxide layers on the surface of stainless steel can give passivity and barrier characteristics. In addition to passivity and barrier qualities, these layers improve corrosion resistance, adhesion, durability, and aesthetics^27^.

Over the past ten years, many Schiff bases have been explored, among other heterocyclic compounds^28^. Additionally, several potential technological uses exist for ligands and their nitrogen and oxygen complexes^29–31^. Amine and a carbonyl group condense to generate a Schiff base, an organic molecule with the generic formula R-C = N-R’, where R and R’ are aryl, alkyl, cycloalkyl, or heterocyclic groups. This can be a possible inhibitor for many metallic surfaces. It has been observed that several Schiff bases effectively inhibit carbon steel corrosion in HCl solutions^32,33^. The information indicates these inhibitors work by adsorption at the metal/solution interface. However, studies on metal complexes as corrosion inhibitors for steels in acidic environments are scarce and have not been widely published^34^. Tomar et al.^35^ concentrate on using a Schiff base (SB), specifically (1Z,4Z)-N′1, N′4-bis(4-methoxyphenyl) succinimidohydrazide (SBMPSH), to prevent mild steel (MS) from corroding in 1.0 M HCl. Abdel-Gaber et al.^36^ identified that during the corrosion inhibition of carbon steel submerged in an H_2_SO_4_ solution, many active sites were covered with large Co (III) Schiff base complex molecules. An azomethine (− CH = N − or > C = N− ) grouping found in many Schiff base compounds makes them effective corrosion inhibitors^33^.

Taha et al.^18^ have studied the corrosion inhibitory characteristics of two Schiff base complexes, Cd(II) and UO_2_(II), including the ligand N-carbamimidoyl-4-((4-chlorobenzylidene)-amino)benzenesulfonamide, on mild steel in hydrochloric acid. Weight loss measurements showed protection efficiencies of 57.56% and 98.48% for Cd(II) and UO_2_(II) complexes at 500 ppm and 25^◦^C, respectively. Baboukani et al.^13^ studied how cobalt (Co) complexes with a Schiff base ligand affect the electrochemical corrosion of 316 L stainless steel in 0.1 M sulfuric acid at 25^◦^C. At a 100 ppm concentration, the inhibition efficiency was 74.4%. The corrosion inhibition of (N1E)-N1,N2-bis(4-(dimethylamino)benzylidene)-ethane-1,2-diamine, DMAB, against C-steel degradation in a dilute HCl environment was investigated^37^. The inhibitory effectiveness rises with concentration, reaching 97.7% at 5.0 mM and 298 K. Tomar et al.^38^ developed and tested schiff bases of 4-methoxybenzyl amine and hydrazide derivatives incorporating succinic (BMSHE), oxalyl (BMOHE), and phenylaceto (BMPHE) to inhibit mild steel corrosion in 1.0 M HCl. All inhibitors effectively prevented MS in HCl (BMSHE-88%, BMOHE-84%, and BMPHE-72%). According to corrosion potentials, BMOHE and BMPHE are mixed inhibitors, but BMSHE is a cathodic inhibitor. Using electrochemical and quantum chemical approaches, two novel eco-friendly Schiff base metal complexes of chromium (Cr) and cobalt (Co) were tested as corrosion inhibitors for carbon steel in 1 M sulfuric acid. The findings revealed that both complexes had mixed-type inhibitory behavior. The inhibition efficiency of the investigated inhibitors varied from 46.5% to 63.7% at 50 ppm and 86.8% to 89.2% for Cr and Co metal complexes at 400 ppm^39^.

A suitable inhibitor should have essential advantages, such as high inhibition efficiency, low price, low toxicity, and easy production^40,41^. Nanocomposite exhibits such advantages in addition to improving the environmental impact. In the last two decades, nanotechnology has been increasingly important in supporting innovative technological advances to manage steel corrosion^41–43^. The researchers also exploited quantum chemical calculations to predict whether chemical structures could be linked to inhibitory effects^44^. In the present work, the corrosion inhibition of 316 L stainless steel in an aggressive mixed acidic medium was scrutinized at different concentrations of our Cu nanocomplex synthesized green inhibitor using various electrochemical measurements and spectroscopic analysis techniques. The purpose is to shed some light on the corrosion inhibition capability of the prepared Cu_2_L_2_ nanocomplex with 1-((E)-(2-mercaptophenylimino) methyl) naphthalen-2-ol Schiff base ligand and to elucidate its mechanism.

## Experimental

### Green synthesis of the nanocomplex

**Fig. 1:**
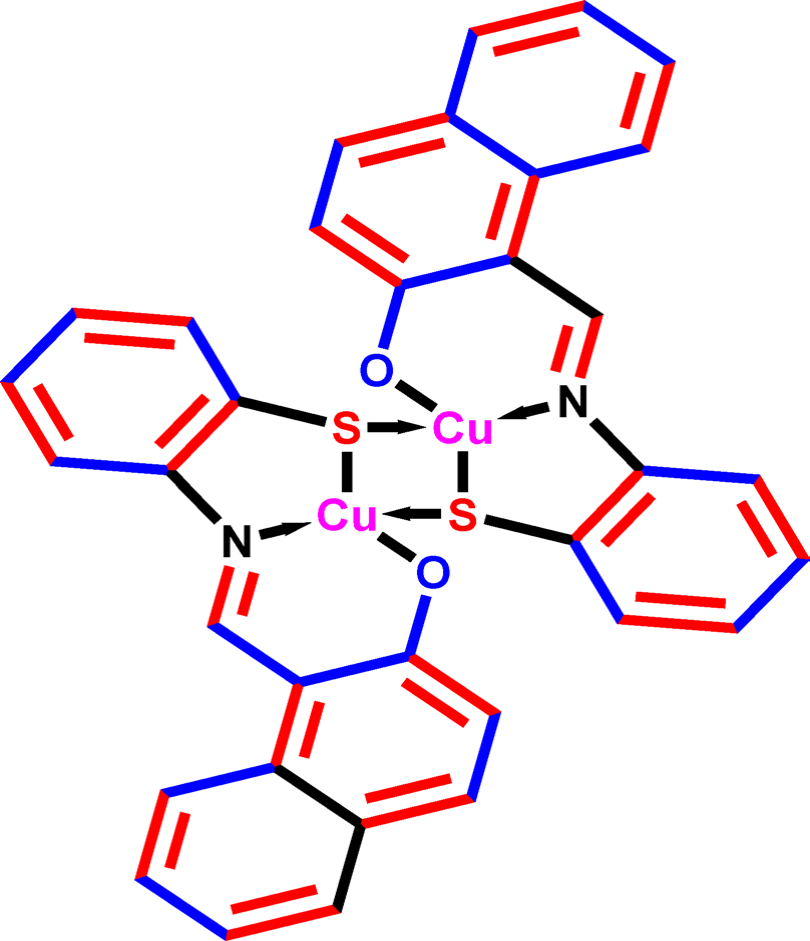
Structure of Cu nanocomplex (Cu_2_L_2_) inhibitor.

**Fig. 2:**
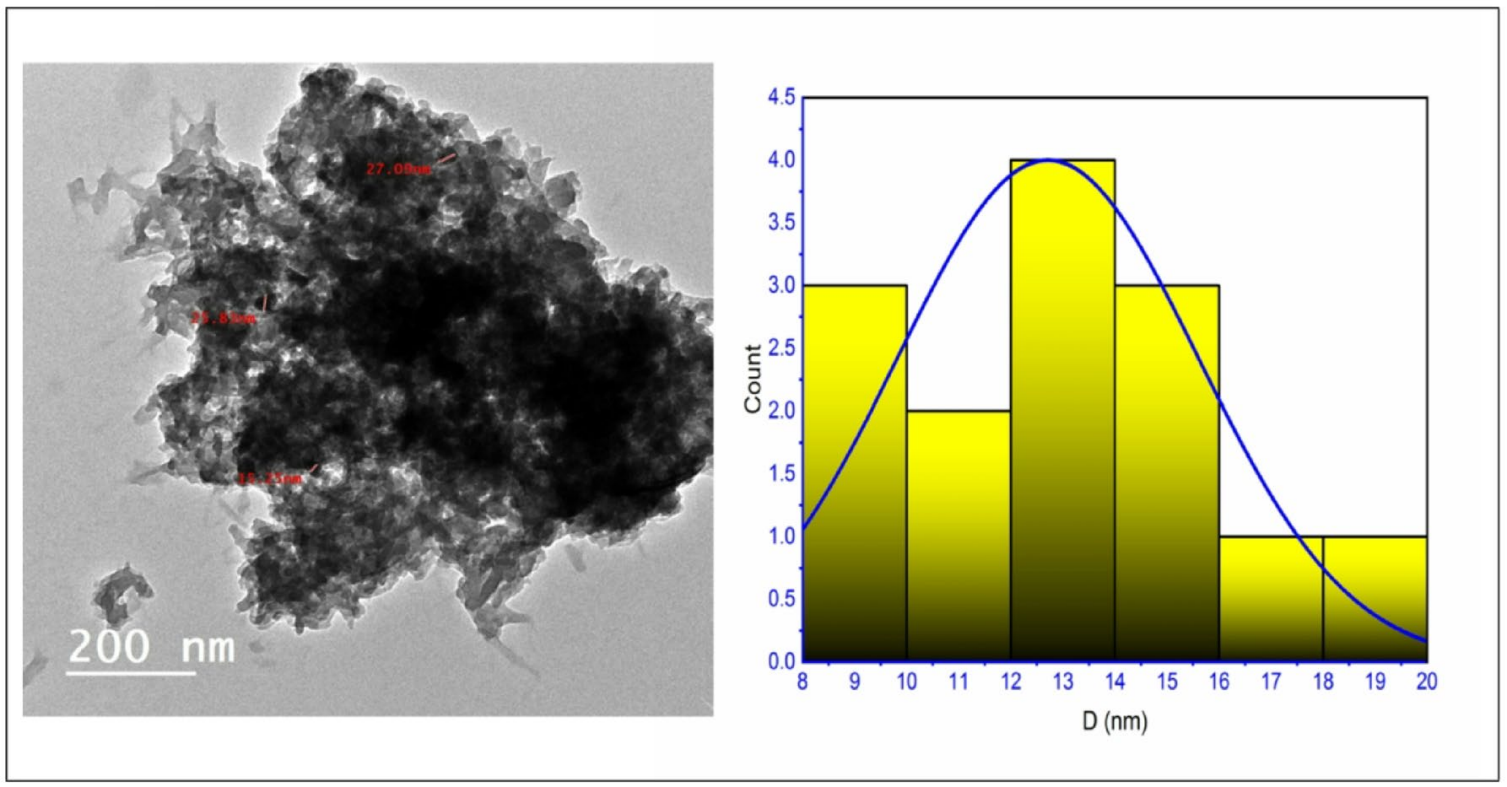
TEM image and histogram of particle size distribution for Cu_2_L_2_ nanocomplex.

As previously specified, the Cu(II) nanocomplex (Cu_2_L_2_) shown in Fig. 1 was synthesized using a solution containing ethanol/*Coriandrum Sativum* (CS)/EtOH with a ratio of 1:1^45,46^. The morphology and size of the Cu nanocomplex created in *Coriandrum Sativum* (CS) media were studied using the TEM technique (Fig. 2). The image demonstrates almost homogeneous cuboid particles, closely related with varying colors on the grey scale. The mean particle size of the Cu nanocomplex was about 22 nm.

### Electrodes and solutions

The WE 316 L stainless steel (SS) has compositions of 0.257% Cu, 0.294% Ti, 0.83% Si, 0.98% Mn, 1.99% Mo, 10.31% Ni, 16.69% Cr, 0.03% C, and balance Fe. The metal sheet was then mounted onto the epoxy resin to offer only one active flat surface exposed to the corrosive environment. These samples were polished mechanically using different grades of emery papers (400, 600, 800, and 1000 grit) and then further sonicated with acetone and alcohol for 15 min to remove all polishing debris. The acids used to prepare the test binary mixture (1 M HCl + 1 M HNO_3_) solution were purchased from Sigma Aldrich, analytical grade chemicals, and bi-distilled water. The extract concentrations were taken in mg per liter (ppm) for all investigations.

### Electrochemical measurements

Open-circuit potential (OCP), electrochemical impedance spectroscopy (EIS), and potentiodynamic polarization were used as electrochemical testing techniques. The measurements were performed in a conventional three-electrode cell assembly with the alloy sample as the working electrode (WE), Ag/AgCl, and a large platinum sheet of size 40 mm × 20 mm × 2 mm as a reference and counter electrode, respectively. The measuring instrument was an electrochemical workstation IM6e Zahner-elektrik, GmbH, Koronach, Germany, controlled by a computer with Thales’ software for I/E and impedance data measurements/analyses. Impedance measurements were recorded at the OCP in the 5 × 104 Hz frequency range to 10^− 2^ Hz using a sinusoidal ac excitation signal of 10 mV peak to peak. In each Cu_2_L_2_ nanocomplex containing electrolyte, three consecutive measurements were taken after 30 min from the sample immersion. EIS data were analyzed and fitted using EC-Lab demo version 11.50 (Bio-Logic^®^).

Tafel polarization log *i* vs. *E* plots were scanned in the potential domain from − 0.45 V to − 0.15 V (vs. Ag/AgCl) using a scan rate of 1 mV s^− 1^. All electrochemical tests were done in stagnant aerated solutions at a room temperature of 25 °C inside an air thermostat. Measurements were performed in triplicate with fresh solutions and newly abraded electrode surfaces to obtain reproducible results.

### Chemical weight loss measurements (WLM)

The 316 L SS was mechanically scraped with 320–1000 grit sandpaper. The samples were then rinsed in double-distilled water, degreased in ethanol, and washed with acetone. Finally, the samples were dried and weighed. To evaluate the reproducibility of data and determine the average to avoid error%, the samples were submerged three times in a glass beaker containing 25 mL of acidic corrosive solution free of inhibitors and with varying inhibitor doses at 25 °C. After adjusting the temperature and completing the immersion time, the alloys were taken out from the test solution, rinsed with distilled water, dried in a moisture-free desiccator, and reweighed to four decimal places using a sensitive analytical balance Model FA 2104 A. Equation 1 calculates the corrosion rate (CR) in millimeters per year as:

1 $$\:\text{C}\text{R}\:\left(\text{m}\text{m}/\text{y}\right)=\frac{\varDelta\:\text{W}\times\:\text{K}}{\text{A}\times\:\text{t}\times\:\text{d}}$$

where K is a constant (8.76$\:\times\:{10}^{4}$), t = time of immersion in days, A = electrode area in cm^2^, ∆W = weight loss in g, and d = electrode density in g/cm^3^.

### Density functional theory calculation

The equilibrium geometry of the complex was studied using DFT calculations at the B3LYP/6-311G+(dp) level of theory, excluding metal ions, performed with the Gaussian 09 program^33^.

### Molecular dynamics simulation

Molecular dynamics (MD) simulations were performed at the molecular level using Forcite quench dynamics to sample many different low-energy configurations and determine the low-energy minima for each inhibitor molecule’s adsorption on the Fe (110) surface using Materials Studio 7.0 software (Accelrys, Inc.). The COMPASS III force field and Smart algorithm were used to do calculations in a 4 × 4 supercell. The Fe crystal was cleaved at the (110) plane. The Fe slab was larger than the inhibitor molecules to avoid edge effects during docking. The temperature was set to 350 K using the NVE ensemble, with a time step of 1 fs and a simulation time of 5 ps. The system was quenched every 250 steps, with the surface atoms of Fe (110) restricted. The simulation approach utilized previously developed inhibitor molecule configurations. Adsorption of a single inhibitor molecule onto the Fe (110) surface provides insight into the adsorption energetics and their impact on the molecule’s inhibitory performance. Equation 2 was used to compute the adsorption energy (E_ads_) and binding energy (BE) between the inhibitor molecule and the Fe (110) surface^47^.

2 $$\:{\text{E}}_{\text{a}\text{d}\text{s}}\:=\:-\:\text{B}\text{E}\:=\:{\text{E}}_{\left(\text{F}\text{e}\:+\:\text{M}\text{o}\text{l}\right)}\:-\:({\text{E}}_{\text{M}\text{o}\text{l}}+\:{\text{E}}_{\text{F}\text{e}})$$

where E_ads_ is the adsorption energy equal to the negative of binding energy, E_ads_ is the energy generated when an inhibitor molecule (adsorbate) adheres to the metal surface Fe (110) (substrate). It contains both stiff adsorption energy and deformation energy. The adsorption energy is the energy released during the inhibitor’s stable adsorption on the Fe (110) surface (also known as the geometric optimization step). In contrast, the deformation energy is released when the adsorbed inhibitor is removed from the Fe (110) surface. E_Fe+Mol_ is the energy corresponding to the combined energies of the Fe (110) surface and the inhibitor molecule, E_Mol_ is the energy of the inhibitor molecule, and E_Fe_ is the energy of Fe.

### Spectroscopic analysis

The morphology and topography characterization were performed using a field emission scanning electron microscope (FE-SEM Quanta FEG 250) attached to an EDX Unit (Energy-dispersive X-ray Analyses). Three 316 L SS samples after 5 days of submerging in binary acid mixtures without and with Cu_2_L_2_ nanocomplex were chosen for the SEM/EDX analysis: (A) blank sample in mixed acid (HCl + HNO_3_), (B) corroded sample in mixed acid containing 100 ppm, and (C) corroded sample in mixed acid containing 600 ppm.

## Results and discussion

### Open circuit potential measurements

**Fig. 3:**
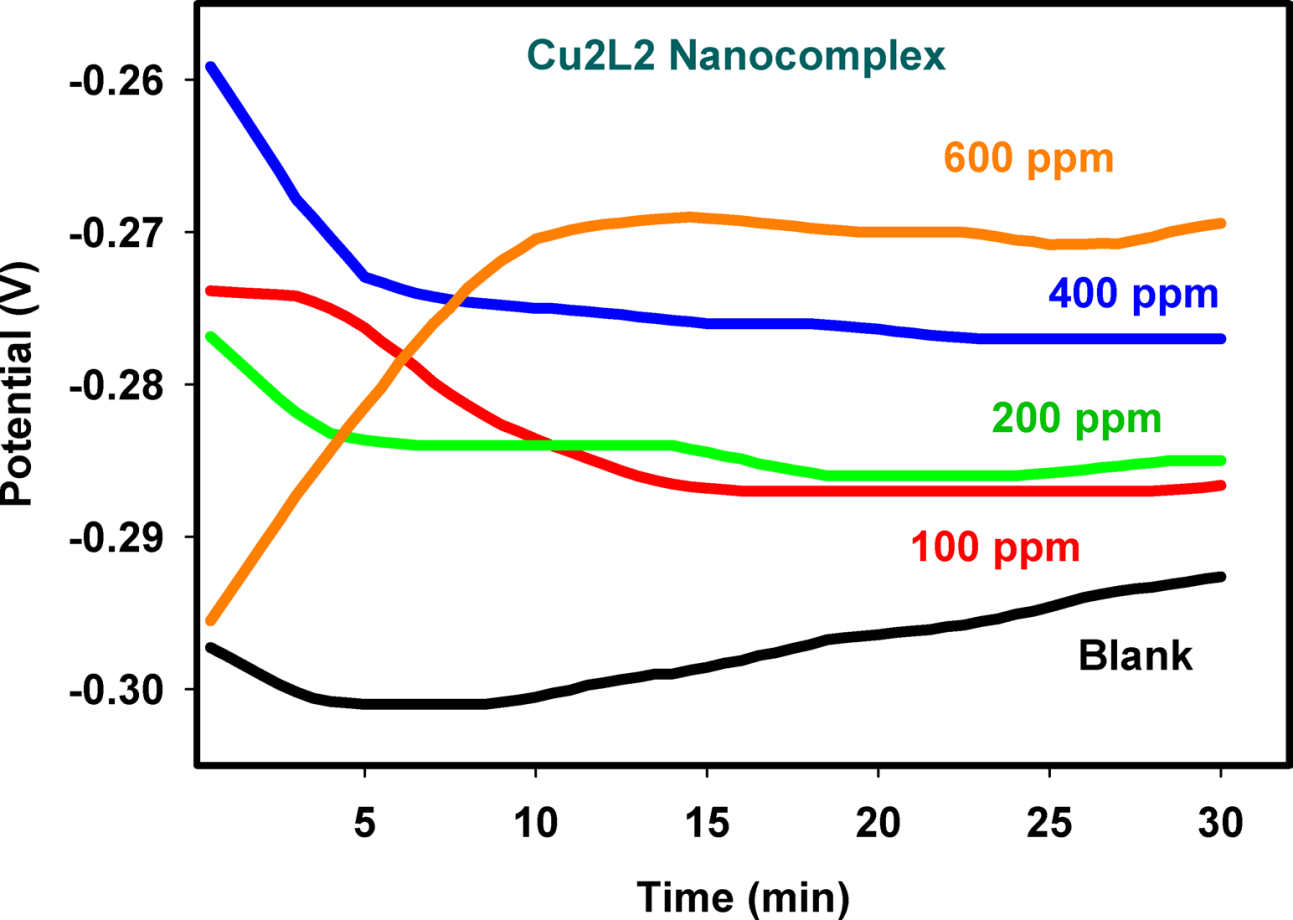
Variation with time of the open circuit potential for the 316 L SS samples in the binary acid mixture without and with different Cu_2_L_2_ nanocomplex concentrations at 25 °C.

The open circuit potential (OCP) of 316 L SS was monitored over 30 min in the binary acid mixture without and with different Cu_2_L_2_ nanocomplex concentrations, namely, 100, 200, 400, and 600 ppm, as shown in Fig. 3. The results can offer essential information about the system’s inherent corrosion behavior under the equilibrium conditions where no external current or potential was applied. Generally, a similar pattern can be seen in all solutions except the one containing 600 ppm during the OCP growth. For the first 5 min, the potential drifts quickly towards more negative values, and then it progressively slows down till reaching a nearly constant value within ~ 15 min. Regardless, after 15 min, the potentials were more stable than they had been at the start for all inhibition concentrations^48^. This behavior displays the air-formed oxide layer’s disintegration mechanism by the acid medium attack. However, at 600 ppm addition, the potential value increases exponentially from the inception of immersion till achieving a constant value after nearly 15 min. The net positive drift in the electrode potential assumes passive film formation due to the Cu_2_L_2_ nanocomplex adsorption that can effectively seal the active anodic sites on the sample surface and hinder its attack. This feature proves that the corrosion inhibition process is under anodic control. Compared with the blank solution, the steady-state potential of the SS sample can be easily achieved in any solution containing Cu_2_L_2_ nanocomplex, with a progressive shift toward more positive values as Cu_2_L_2_ concentration is increased in the corrosive media. This conduct can arise from the Cu_2_L_2_ nanocomplex adsorption on the metal surface, leading to its passivation^49^. Physical adsorption occurs due to the interaction between positive charges on the Cu_2_L_2_ nanocomplex and the negative charges on the submerged steel surface. As a result, the potential drifts at any interval > 15 min towards a more positive steady-state value with increasing the inhibitor amount in the corrodent. In any case, it is further seen from the results of the OCP measurements in Fig. 3 that the positive shift in the steady potential value is too small for a sensible dose addition and becomes more significant for higher concentrations.

Although OCP was monitored for 30 min in the main experiments, a representative long-term measurement (90 min at 400 ppm) was also performed. The result (Fig. S1) confirmed that extending immersion beyond 30 min did not materially alter the steady potential, thus validating the selected immersion time.

### EIS measurements

**Fig. 4:**
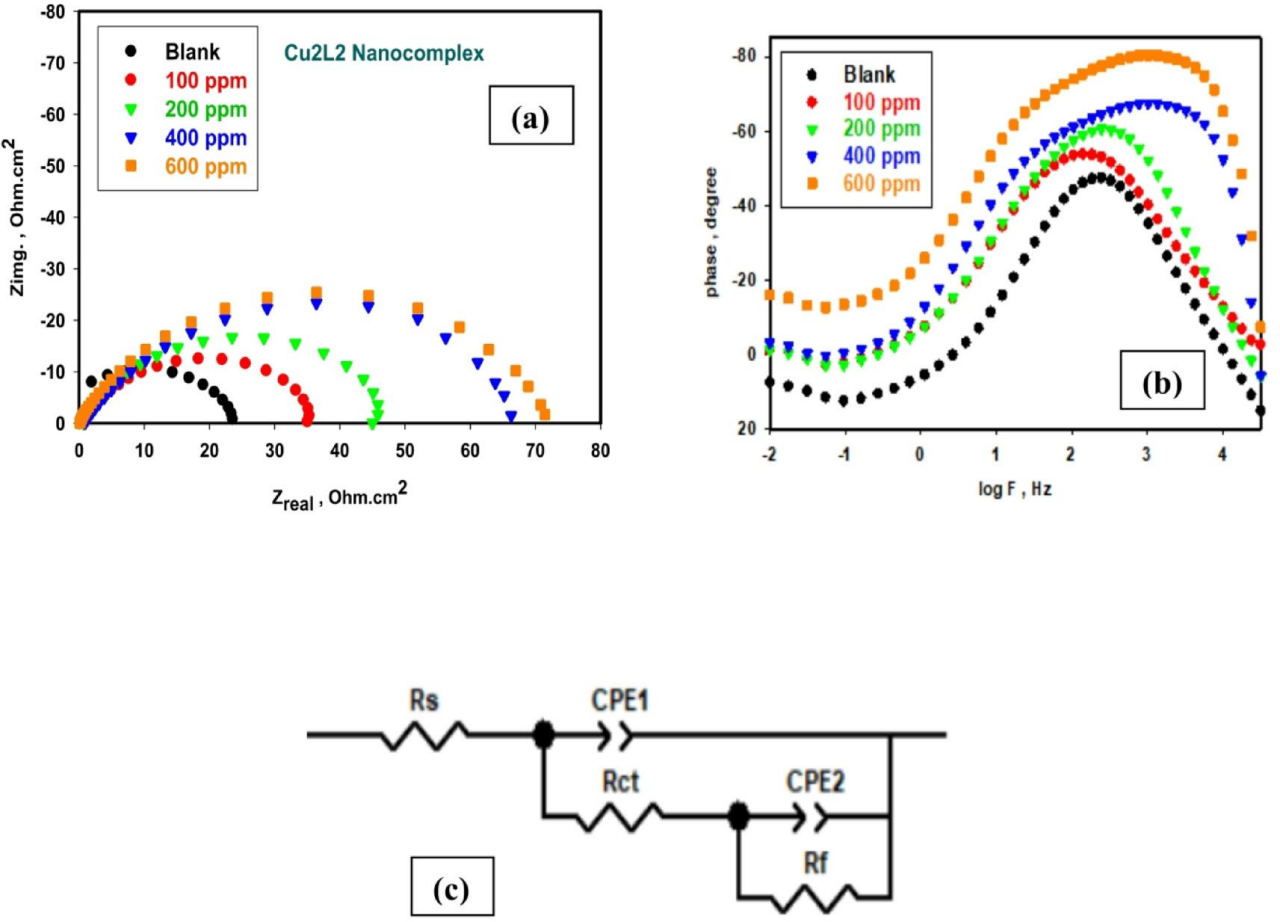
EIS spectra (**a**) Nyquist plots, (**b**) Bode phase angle plots, and (**c**) the EC used to fit the measured EIS spectra of 316 L SS in the binary acid mixture without and with different Cu_2_L_2_ nanocomplex concentrations at 25 °C.

EIS is a non-destructive method that provides valuable information about surface properties and the efficiency of the inhibitor. The EIS results, as Nyquist and Bode (phase-log f) plots for 316 L SS in the binary acid mixture without and with different Cu_2_L_2_ nanocomplex concentrations recorded at the OCP after 30 min of immersion, are presented in Fig. 4a, b. The Nyquist plots shown in Fig. 4a all have a semicircle shape within the high to medium frequency range, with a diameter that increases as the Cu_2_L_2_ nanocomplex dose in the medium increases. This half-circle loop is credited to the double-layer capacitance and charge transfer control^23,50^ that ended with another loop at the low-frequency range, especially in solutions containing higher inhibitor concentrations, as shown by the Bode phase angle plot in Fig. 4b. The impedance spectra are not an ideal half-circle but a depressed capacitive loop compared with the surface heterogeneity and roughness due to the corrosion and surface adsorption of the inhibitor molecules^51–53^. The charge transfer resistance value increases with the increase in Cu_2_L_2_ nanocomplex concentration. Its value measures how facile electrons transfer across the interface, which is inversely proportional to the corrosion rate^54^. The nature of the Nyquist plots is similar in the uninhibited and inhibited solutions, demonstrating that Cu_2_L_2_ nanocomplex inhibitor does not change the corrosion mechanism of the steel sample. The most suitable equivalent circuit (EC) used to fit the experimental impedance spectra was found to be the two-time constant model shown in Fig. 4c. The EC consists of a solution resistance (R_s_) in series with a parallel combination of the charge transfer resistance, *R*_ct_, and a double-layer capacitor (CPE1) representing the electrode capacitance behavior^55^. The second time constant is the parallel combination of the CFE2 and the resistor (*R*_f_), expressing the property of the surface passive film. It is noted that R_s_ has a value that is too small compared to *R*_ct_ and hence can be ignored.

The EIS parameters are listed in Table 1. From the *R*_ct_ values, the percentage inhibition efficiency (IE%) was estimated utilizing Eq. 3 below^33^. The IE% increases in a way consistent with the amount of Cu_2_L_2_ nanocomplex in the solution. The results obtained from EIS are congruent with those of OCP, generally indicating the efficacy of Cu_2_L_2_ nanocomplex as a potential corrosion inhibitor for SS in the aggressive binary acid mixture (1 M HCl + 1 M HNO_3_) solution.

3 $$IE {\%}\:=\:\left(1-\frac{{R}_{ct}^{o}}{{R}_{ct}}\right)\times\:100$$

*R*^o^_ct_ and *R*_ct_ are the charge transfer resistance obtained in the blank and inhibited solutions.

**Fig. 5:**
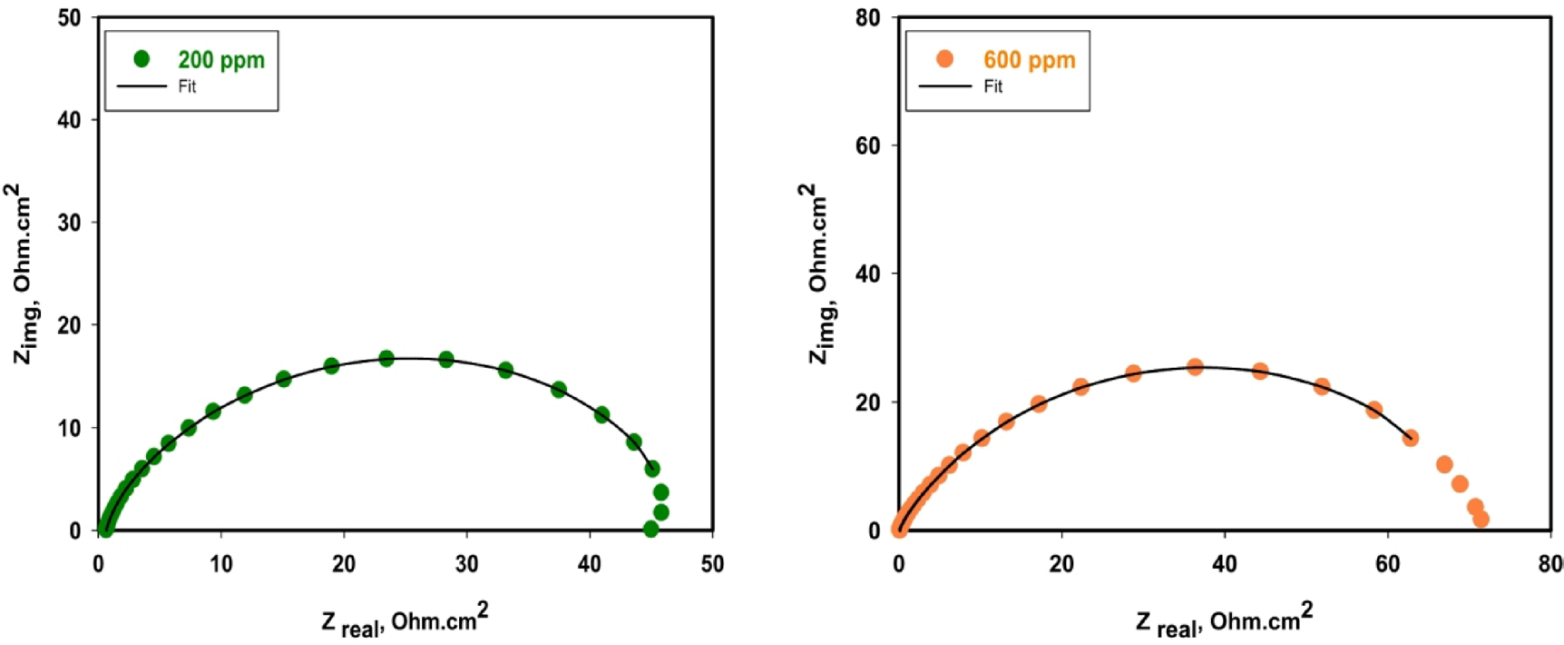
The fitting curve for 316 L SS in the binary acid mixture containing Cu_2_L_2_ nanocomplex.

Figure 5 depicts the fitting curves for 316 L SS in a binary acid mixture containing the Cu_2_L_2_ nanocomplex. Table 1 reveals that *R*_ct_ increases while CPE₁ decreases with increasing inhibitor concentration. This trend indicates that when nanocomplex Cu₂L₂ adsorbs on the 316 L SS, it blocks active sites and leads to slower charge transfer. The Cu₂L₂ nanocomplex successfully inhibits 316 L SS corrosion in (HCl + HNO₃) medium with progressively increased efficiency up to 84.15% at a concentration of 600 ppm, giving the highest R_ct_ value.

The obtained values of *R*_ct_ and *R*_f_ were relatively close, highlighting the dual contribution of both charge transfer and film resistance to the inhibition process. While *R*_ct_ is considered the main parameter for calculating inhibition efficiency, the comparable values of *R*_f_ confirm the effective formation of a protective surface layer. This indicates that the nanocomplex inhibits corrosion through a mixed mechanism, combining surface film formation with suppression of charge transfer reactions.

It is plausible that HNO_3_ could cause passivation of stainless steel, leading to a two-time-constant model. Table 1 displays the chi-square (χ^2^) results that validate the proposed circuit’s fit. Smaller χ^2^ values indicate a better match of the recommended EC, which aligns with the experimental evidence. The error percentages obtained from fitting (Table 1) appear slightly higher due to the complexity of the Cu_2_L_2_ nanocomplex in the aggressive (HCl + HNO₃) medium, the non-ideal capacitive response of the interface, and slight instabilities during long-term measurements in mixed acid solution.

**Table 1:** EC impedance parameters and corresponding Inhibition efficiency for 316 L SS in the binary acid mixture without and with different Cu_2_L_2_ nanocomplex concentrations at 25 °C.

System	Conc. (ppm)	*R*_ct (Orig)_ Ω cm²	*R*_ct (Fit)_ Ω cm²	CPE1 µF/cm²	*R*_f_ Ω cm²	CPE2 µF/cm²	X^2^	Error %	IE%
**Blank (HCl + HNO**_**3**_ **)**	0.0	13.14	12.1389	1.8016	21.1006	0.90662	0.3800	11.076	--
**+ Cu**_**2**_ **L**_**2**_ **nanocomplex**	100	34.72	13.8961	0.5362	26.2364	0.80886	1.5474	07.596	62.15
	200	42.21	18.8224	0.3036	58.3770	0.45002	2.2631	17.983	68.87
	400	65.25	72.7359	0.1068	93.5316	0.39758	2.6277	21.730	79.86
	600	82.89	91.2594	0.0328	97.1864	0.39418	3.1132	22.985	84.15

### Potentiodynamic polarization (PDP) measurements

**Fig. 6:**
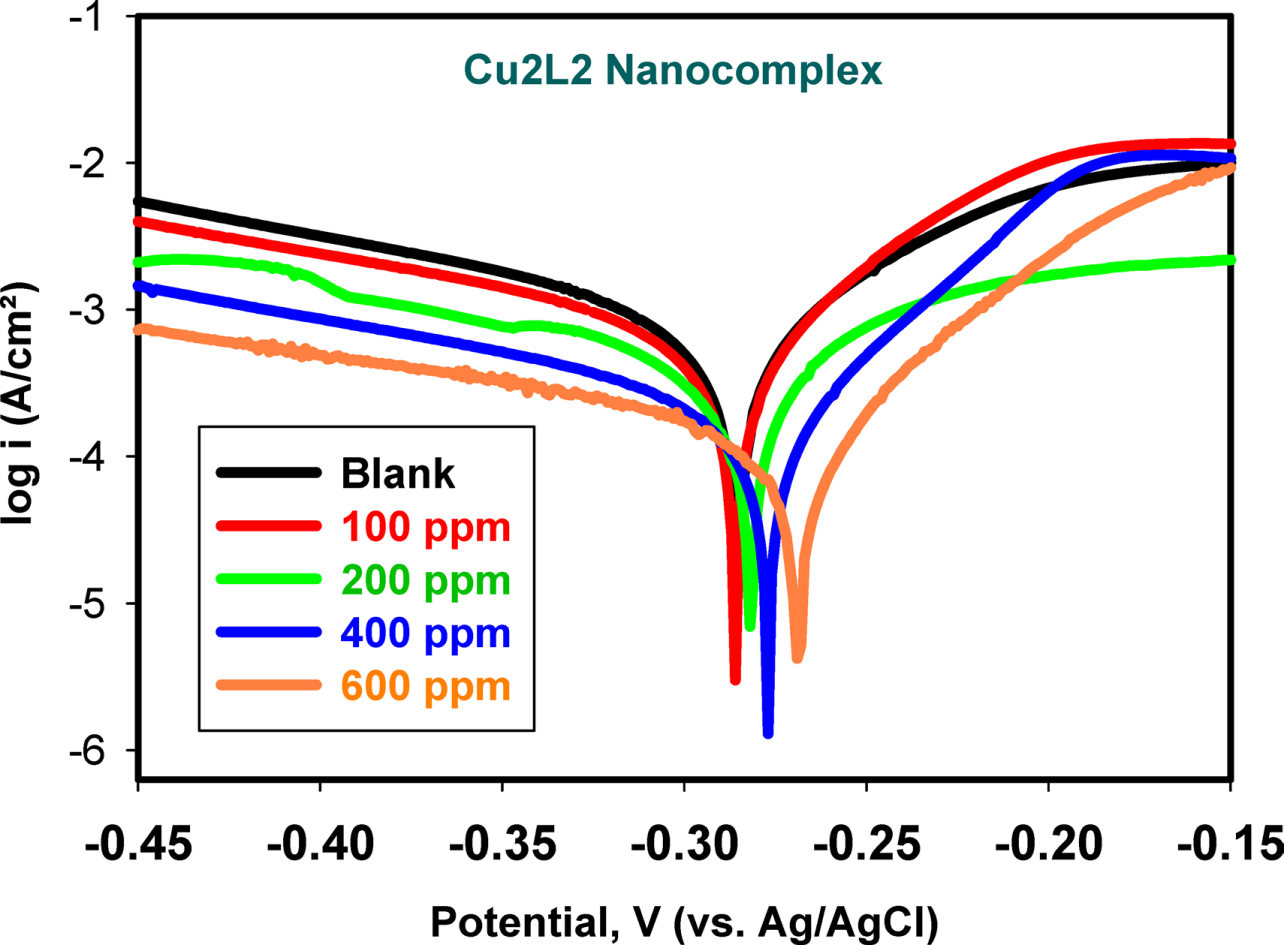
Potentiodynamic polarization curves of 316 L SS in the binary acid mixture without and with different concentrations of Cu_2_L_2_ nanocomplex at 25 °C.

Figure 6 shows the PDP curves of SS samples in the corrosive media without and with different concentrations of the Cu_2_L_2_ nanocomplex. The cathodic and anodic Tafel lines were analyzed to derive the various corrosion parameters, such as corrosion potential (*E*_corr_), anodic and cathodic Tafel slopes (*β*_a_ and *β*_c_), corrosion current density (*i*_corr_), and the standard deviation (S.D.), as shown in Table 2. The percentage inhibition efficiency (*IE*%) values derived from Eq. 4 below are also listed in this Table

4 $$\:IE {\%}\:=\:(1\:-\:\frac{i}{{i}^{o}}\:)\:\times\:\:100$$

*i* ^o^ and *i* are the values of the corrosion current densities obtained in the blank and inhibited solutions. The corrosion rate (*CR*) was calculated from Eq. 5 as follows^56^:

5 $$\:CR\:\left(\frac{\text{m}\text{m}}{\text{y}\text{e}\text{a}\text{r}}\right)=\:3.27\:{i}_{corr}\:\text{M}/\text{z}\:\text{d}$$

where z is the ionic charge (= 2), M is the metal’s atomic weight (= 55.845 g/mol), d is the iron density (= 7.86 g/cm^3^), and *i*_corr_ is the corrosion current density (in mA/cm^2^).

The surface coverage (*θ*) is thus equal to (% *IE/*100)^57^. As can be seen, the inhibitor Cu_2_L_2_ nanocomplex lowers the cathodic Tafel branch without changing its slope, significantly affecting the anodic one. In addition, the *E*_corr_ value moved toward more negative values relative to the blank by incorporating a larger amount of Cu_2_L_2_ nanocomplex in the solution. It has been established that an inhibitor to be considered cathodic or anodic requires a shift of ± 85 mV in the *E*_corr_ between the blank and inhibited systems; otherwise, it is classified as a mixed-type one^57^. The *E*_corr_ values in Table 3 confirm that the Cu_2_L_2_ nanocomplex is of the mixed-type category^58,59^. The higher the concentration of the Cu_2_L_2_ nanocomplex is, the lower the corrosion rate. This result can be explained by the increased inhibitor protection affinity parallel to the upsurge in adsorption of Cu_2_L_2_ molecules on the SS surface at higher concentrations. Due to the reverse relationship between *R*_p_ and *i*_corr_ through expanding the concentration of Cu_2_L_2_ nanocomplex inhibitor, it is commonly assumed that the adsorption of Cu_2_L_2_ molecules takes place on the metal surfaces, creating physical and charge-transfer barriers to protect the metal surfaces to a high degree. The inhibition activity increased with concentration till achieving 85.56% at 600 ppm. The reduction in the corrosion current density and the development in the hindrance efficacy point to the adsorption of the investigated Cu_2_L_2_ nanocomplex compound on the sample surface. It is worth mentioning that the Tafel polarization tests shown in Fig. 6 confirm the EIS ones in Fig. 4, revealing that the corrosion rate decreases dramatically in the presence of Cu_2_L_2_ inhibitor at all concentrations studied compared to the blank. The anodic and cathodic reactions are thus significantly hampered. At 200 ppm, the potentiodynamic polarization curve exhibited a slightly different behavior than the other concentrations. This can be attributed to the competitive adsorption and partial saturation of inhibitor molecules on the metal surface at this concentration. As a result, the inhibition efficiency reached a transition state, leading to a deviation in the anodic/cathodic branch slopes.

Although inhibitors can disrupt cathodic and anodic processes, anodic reactions are more affected. The anodic Tafel slope (*β*_a_) falls compared to the blank (130.8 mV/dec) at lower concentrations (75.9, 85.3 mV/dec at 100 & 200 ppm) and slightly decreases at the higher concentration (69.0 mV/dec at 600 ppm). The total decrease indicates that the inhibitor alters the anodic dissolution mechanism mainly by generating a blocking layer for their active sites on the SS surface. The Cathodic slope (*β*_c_) reveals some slight alteration in the inhibited solutions compared to the blank.

**Table 2:** Polarization corrosion parameters and the Inhibition efficiency for 316 L SS in the binary acid mixture without and with different Cu_2_L_2_ nanocomplex concentrations at 25 °C.

System	Conc. (ppm)	-E_corr_ mV(vs. Ag/AgCl)	i_corr_ mA/cm^2^	β_a_ mV/dec	-β_c_ mV/dec	CR mm/y	S.D.	Surface coverage (θ)	IE%
**Blank (HCl + HNO**_**3**_ **)**	0.0	286.0	1.5460	130.8	209.1	19.1587	3.215 × 10^− 3^	--	--
**+ Cu**_**2**_**L**_**2**_ **Nanocomplex**	100	282.3	0.4752	75.9	209.3	5.8889	1.154 × 10^− 4^	0.6926	69.26
	200	273.4	0.4357	85.3	251.1	5.3994	3.786 × 10^− 4^	0.7182	71.82
	400	271.5	0.3079	64.4	214.4	3.8156	1.732 × 10^− 4^	0.8008	80.08
	600	269.0	0.2232	69.0	224.7	2.7660	1.155 × 10^− 4^	0.8556	85.56

### Effect of immersion time

**Fig. 7:**
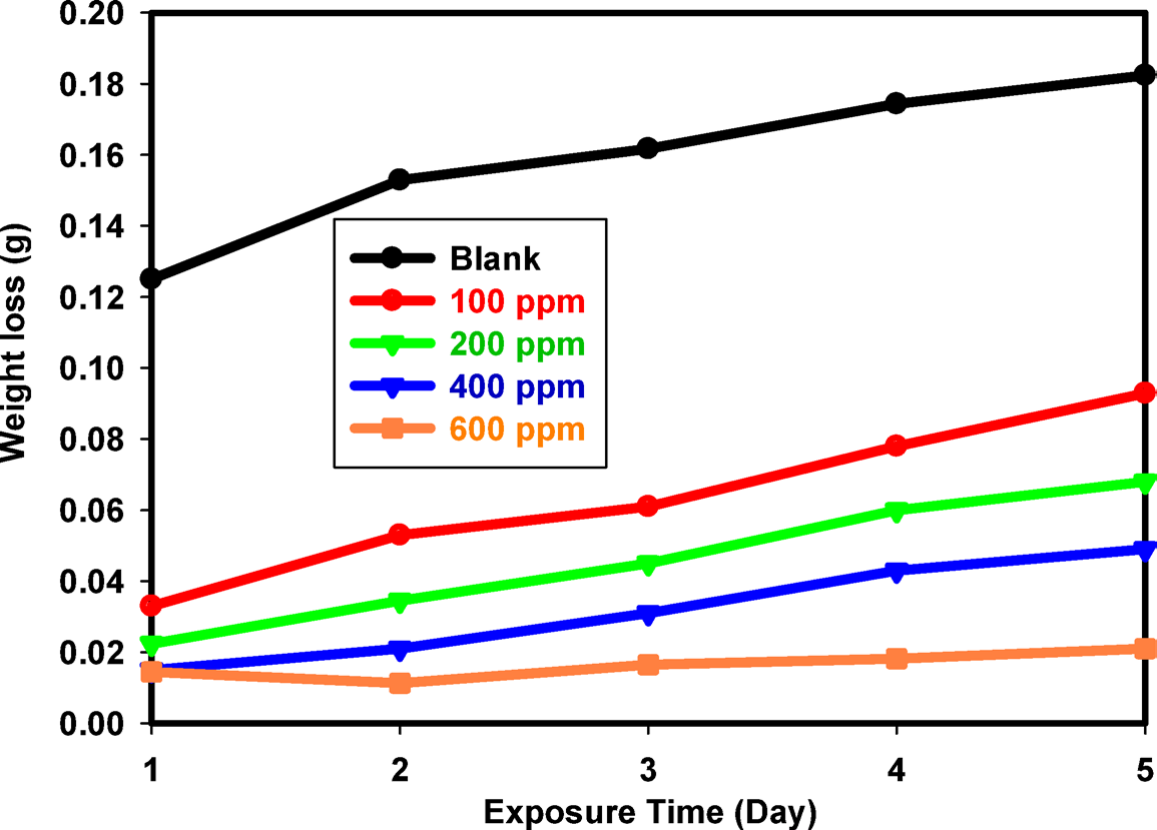
Weight loss-time plots for the corrosion of 316 L SS samples in the binary.

Figure 7 displays the impact of immersion time on the weight loss of 316 L SS in the binary acid mixture without and with different Cu_2_L_2_ nanocomplex concentrations at 25 °C. The loss in sample weight gradually increased with prolonging the immersion duration at the same inhibitor dose. Meanwhile, at any given immersion period, the WL decreased with increasing inhibitor concentration, indicating a corresponding increase in the amount of the inhibitor adsorbed on the sample surface. Raising the inhibitor concentration prone to more functional groups improves the protection efficiency due to the continual creation of a better protective layer on the SS surface.

**Fig. 8:**
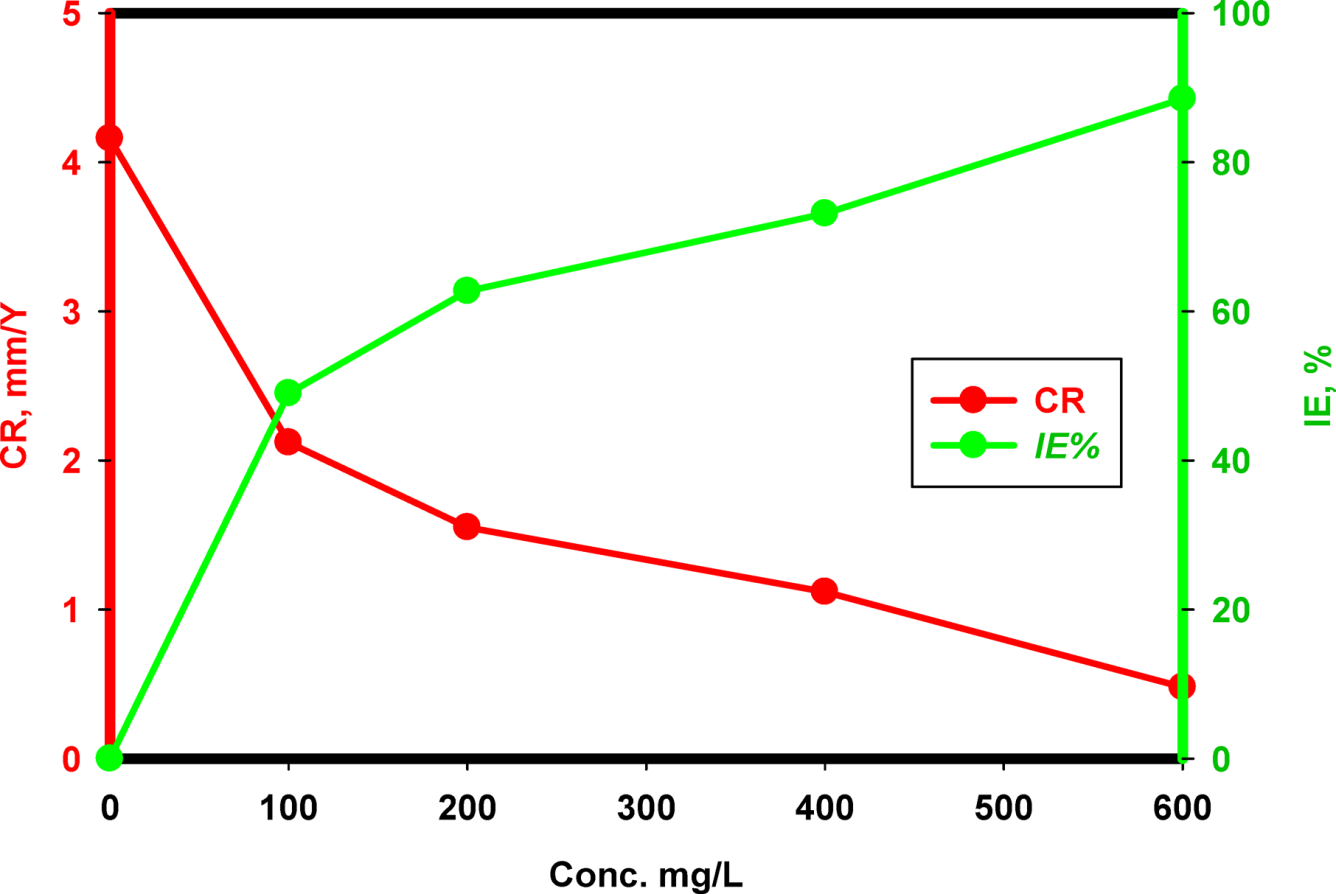
The variation of corrosion rate and inhibition efficiency on the 316 L SS in the binary acid mixture without and with different Cu_2_L_2_ nanocomplex concentrations at 25 °C.

Figure 8 shows the variation with time of the corrosion rate (*CR*) and the inhibition efficiency (*IE*%) values evaluated from WL for the SS samples over 5 days of immersion in binary acid solution. The corrosion rate is relatively high at 0 mg/L (blank), with a value of 4.1587 mm/year. Corrosion rates drop dramatically as Cu₂L₂ concentration grows. At 600 mg/L, the corrosion rate falls below 0.47906 mm/year compared to the other concentrations 100, 200, and 400 mg/L, at which the *CR* were 2.11928, 1.55125, and 1.117812 mm/year, respectively, validating that nanocomplex Cu₂L₂ efficiently inhibits the corrosion of 316 L SS under corrosive conditions. The results confirmed that the best protection efficiency of 88.5% was at 600 mg/L from the studied inhibitor, compared to 100, 200, and 400 mg/L, which gave values of 49.0%, 62.7%, and 73.1%, respectively. To conclude, Cu₂L₂ nanocomplex does inhibit 316 L SS corrosion in a binary acid combination at 25 °C, and the corrosion rate reduces with increasing nanocomplex concentration, suggesting lower material deterioration. At the same time, inhibition efficiency improves, indicating improved protective activity.

acid mixture without and with different Cu_2_L_2_ nanocomplex concentrations at 25 °C.

### DFT parameters and inhibitor efficiency relationships

**Fig. 9:**
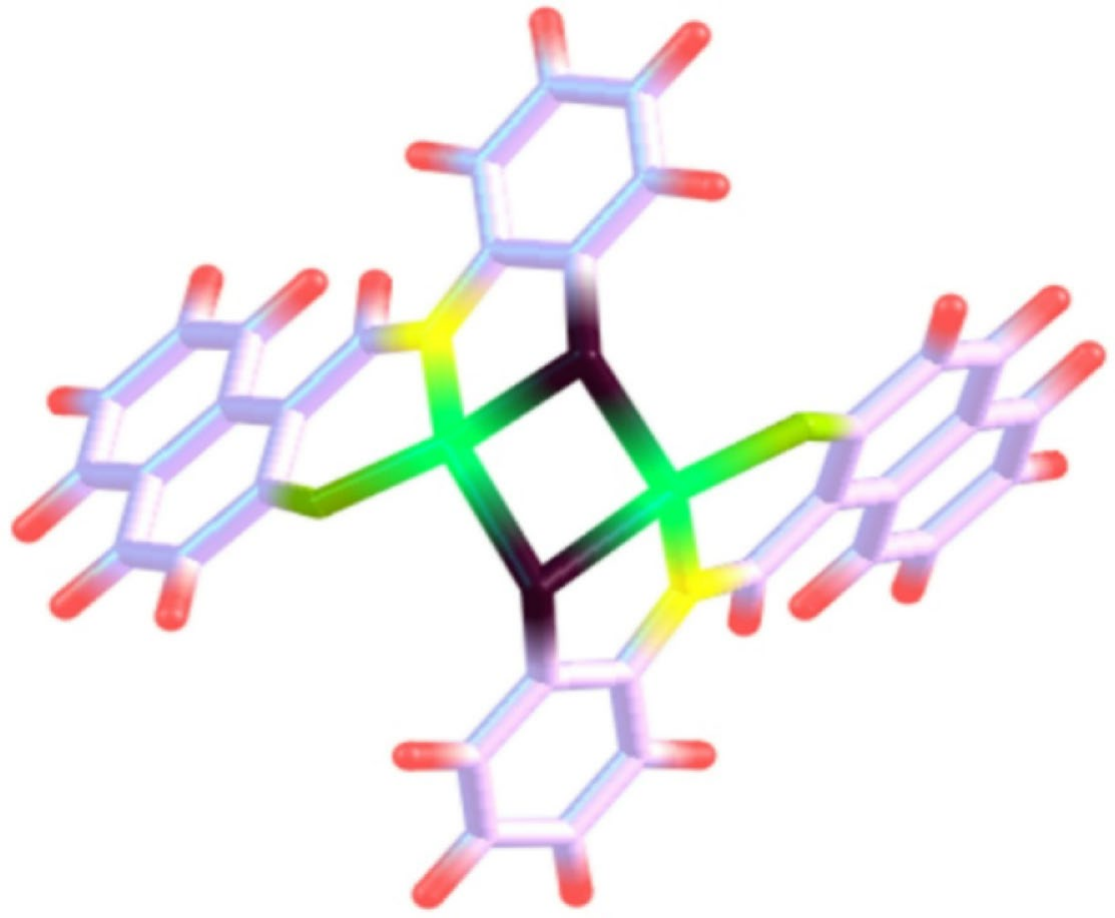
Optimized structure of Cu_2_L_2_ nanocomplex inhibitor using the Gaussian 09 program.

A theoretical study is often performed to emphasize the corrosion mitigation mechanism. The more common one is the density functional theory (DFT), among other functions, which appears promising for revealing changes in the electronic structure responsible for the inhibitory action of compounds on metallic surfaces. To further assess its proficiency, a theoretical study using the DFT approach was accomplished on our synthesized Cu nanocomplex as a green inhibitor (Fig. 9). Indeed, the inhibitor’s efficiency depends on its molecular construction.

**Fig. 10:**
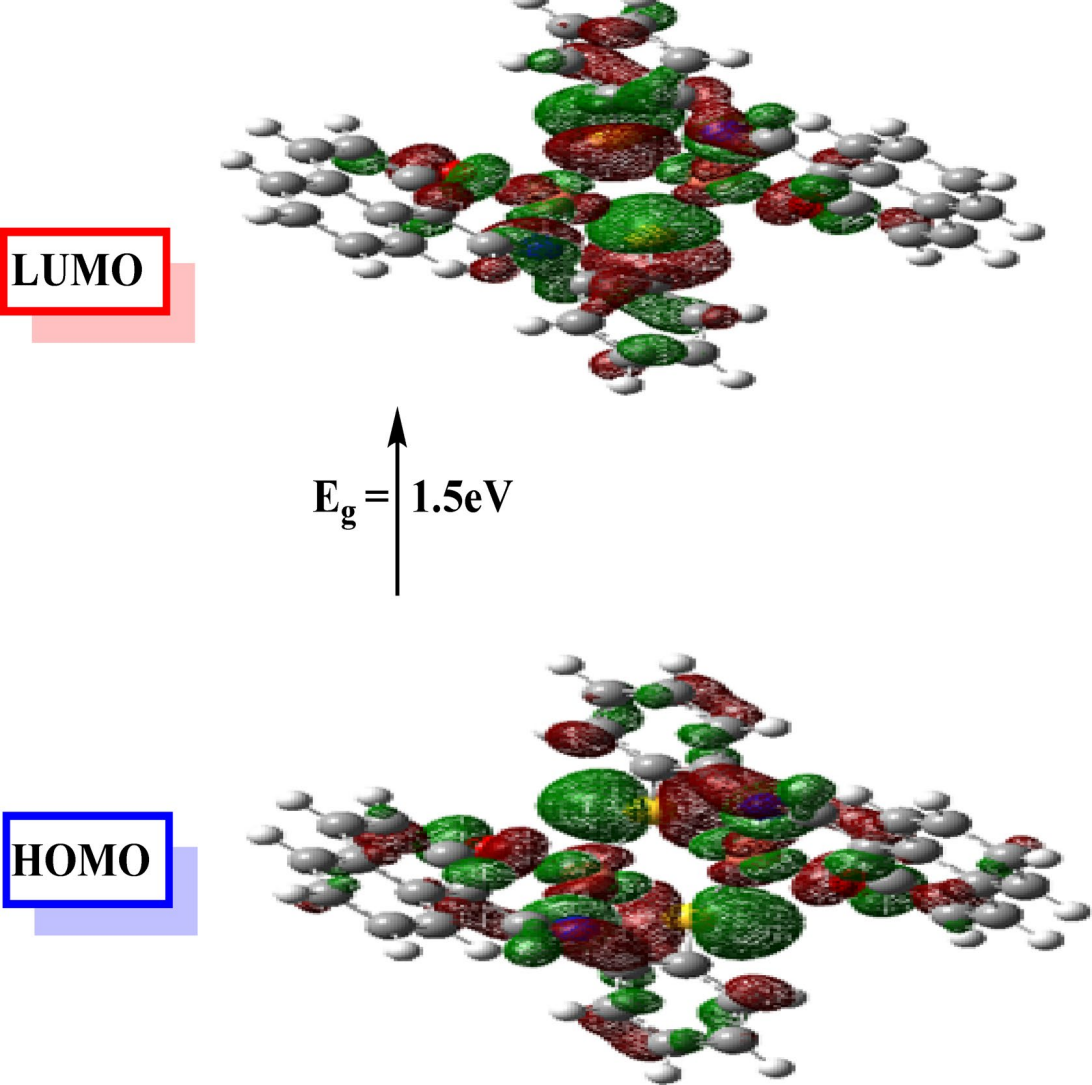
MO and their energies for Cu_2_L_2_ nanocomplex inhibitor using the Gaussian 09 program.

The optimized molecular geometry of the inhibitor Cu_2_L_2_, derived from DFT calculations at the B3LYP/6-31G(d) level, exhibits a tetrahedral configuration surrounding the central atom. Despite the non-planar geometry, the spatial arrangement of active donor atoms (such as N, S, and O), which permits partial adsorption and contact with the metal surface via numerous coordination sites, supports adequate surface coverage and inhibitory efficacy. The frontier orbital model predicts the adsorption centers on the additive compounds, which can interact with the iron atom. As stated before, an efficient inhibitor would contribute electrons to an empty orbital of the metal and accept electrons from the 316 stainless steel atoms at its interface. Therefore, it is crucial to evaluate the energy of the highest occupied molecular orbital (*E*_HOMO_) and the energy of the lowest unoccupied molecular orbital (*E*_LUMO_), the ionization potential (*I* = -*E*_HOMO_), and the electron affinity (*A* = -*E*_LUMO_), as well as the chemical potential (*µ*). The absolute hardness (*η* = -½(*E*_HOMO_-*E*_LUMO_)) reflects the resistance to a charge transfer, while the absolute softness (*σ* = 1/*η*) describes the ability to receive electrons. Those parameters were all calculated for the prepared nanocomplex to validate its mitigation mechanism. Electronegativity (*X* = -½(*E*_HOMO_+*E*_LUMO_)), which is the power of an atom to attract electrons towards itself, was also calculated (Table 3). Inhibitor additives with a high *E*_HOMO_ level easily donate electrons to the vacant acceptor orbitals; meanwhile, the *E*_LUMO_ contributes to the capability of the inhibitor molecule to receive an electron^60^. Figure 10 displays the geometrical constructions with LUMO and HOMO locations. Adsorption of inhibitor molecules on the 316 stainless steel interfaces can thus be completed via two methods. The first is that the additive compound contributes electrons to empty d-orbitals of the iron surface to produce a coordinate bond, and the second is that the additive compound receives electrons from the iron surface atom, which creates a back-bond between the inhibitor and iron atom. It was earlier revealed that the inferior energy gap value (Δ*E* = *E*_HOMO_ - *E*_LUMO_), the greater the protection efficacy, because the energy required for separating an electron from the HOMO orbital is small. The percentage inhibitory efficiency grew as Δ*E* decreased. The *E*_HOMO_, *E*_LUMO_, and Δ*E* results indicate that Cu nanocomplex has a somewhat better capability to suppress corrosion^33^. Several other parameters, including global hardness (*η*), global softness (*σ*), electronegativity (*X*), chemical potential (*µ*), global electrophilicity (*ω*), the fraction of electron transfer (Δ*N*), and energy associated with a back donation (*E*_b−d_), are listed in Table 3 to provide additional insight into the interaction between the inhibitor molecule and the metal surface. Moreover, our work has already given the connections used in these computations^61^.

**Fig. 11:**
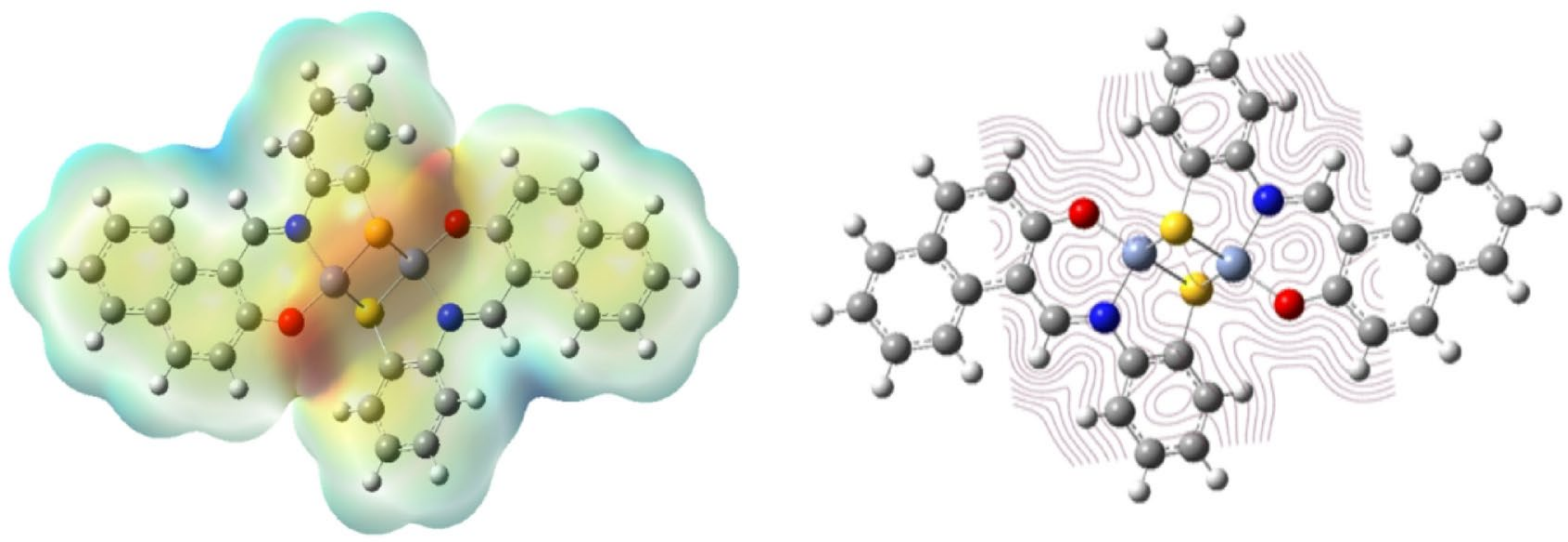
Molecular electrostatic potential (MEP) surface and counterplots for the Cu_2_L_2_ nanocomplex inhibitor, using the Gaussian 09 program.

Inhibitors with low global hardness and high softness values usually exhibit strong chemical reactivity and high inhibitory effectiveness^12^. Based on the results of Table 3, the chemical potential (*µ*) of the nanocomplex is negative, indicating its stability. Also, the Cu nanocomplex’s lower *η* and excellent *σ* values suggest the nanocomplex is more likely to be adsorbed on the metal substrate. To measure a molecule’s ability to absorb or transfer electrons to or from a metal, the proportion of electron transfer (Δ*N*) is used^55^. If Δ*N* > 0, the inhibitor might give its electron to the metal, and the opposite is true if Δ*N* < 0^62^. Thus, it can be inferred from the positive values of Δ*N* for the investigated inhibitor that electron donation occurs from the inhibitor to the metal surface. Back donation from the metal to the inhibitor is energetically beneficial, as revealed by the negative sign of *E*_b−d_. The back contribution and donation procedures promote inhibitors’ adsorption on the iron surface. These findings are consistent with the experimental inhibition effectiveness of the Cu nanocomplex. Predicting the reactivity of inhibitor compounds and the overall distribution of charges can be further done by utilizing the molecular electrostatic potential (MEP)^56^, as shown in Fig. 11. The blue (positive) areas in the resulting synthesized Cu nanocomplex represent the love electron sites. In contrast, the red portions describe the attack on the love nucleus (Fig. 11).

**Table 3:** Quantum chemical parameters for Cu_2_L_2_ nanocomplex inhibitor.

Parameters	Cu nanocomplex
Total energy (Hartree)	-1981.24
Dipole moment (Debye)	6.957
Chemical potential (eV)	− 4.665
electronegativity *X* (eV)	-4.665
*E*_HOMO_ (eV)	-5.415
*E*_LUMO_ (eV)	-3.915
Δ*E* (eV)	-1.5
*η* (eV)	0.75
σ (eV^− 1^)	1.333
∆*N*	1.555
*ω*	14.5081
*E*_b−d_ (eV)	-0.375

### Molecular dynamics (MD) simulations

**Fig. 12:**
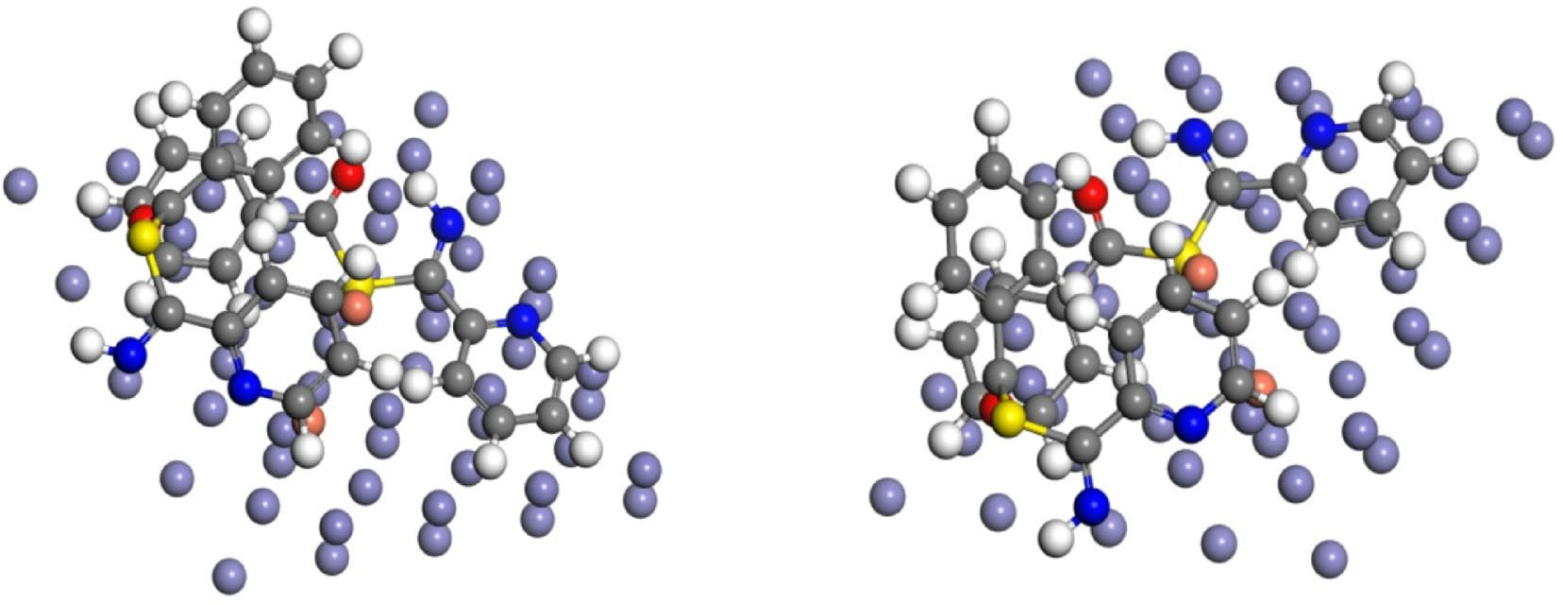
Equilibrium adsorption configurations of the studied Cu_2_L_2_ nanocomplex adsorption.

At the molecular level, MD simulations were performed using Forcite quench dynamics to sample many distinct low-energy configurations to discover the low-energy minima for each examined molecule’s adsorption on the metal surface. The total energies were estimated by averaging the energies of each molecule’s five most stable typical adsorption configurations. Figure 12 shows a side-view snapshot of the lowest energy adsorption configurations for a single inhibitor molecule interacting with the Fe (110) surface. The results show that each inhibitor molecule maintained a flat-lying adsorption orientation on the Fe (110) surface. This placement is helped by the development of bonds between the inhibitor and the Fe surface, which are created through the transfer of p electrons from the inhibitor’s active donor sites to the empty orbitals of the positively charged Fe surface. This adsorption arrangement maximizes the molecule’s interaction with the metal surface. Table 4 shows the adsorption energies obtained for each molecule’s interaction with the Fe (110) surface using Forcite Quench Dynamics. It has been found that the more negative the Eads of the inhibitor-metal surface contact are, the greater the adsorption or binding of the inhibitor onto the metal surface and, thus, the higher the molecule’s inhibitory efficacy. Based on the *E*_ads_ or BE values obtained, the inhibitor with a higher negative value of *E*_ads_ (-291.6 kcal mol^− 1^) is projected to perform better in inhibiting Fe corrosion. This feature may be related to the order of molecular size, with larger molecules more strongly adsorbed on the metal surface. The calculated *E*_ads_ value is less than 100 kcal mol^− 1^, although the simulations did not account for the unique covalent interactions between the molecules and the Fe surface. This observation can be attributed to their molecular size and the whole molecule adsorbed with a flat orientation on the Fe (110) surface^47,63^.

on Fe (110) surface obtained by molecular dynamics simulations’ top and side views, using Materials Studio 7.0 software (Accelrys, Inc.).

**Table 4:** Molecular dynamic simulations of Cu_2_L_2_ nanocomplex adsorption on Fe (110) surface (energies are given in Kcal mol^− 1^).

Total energy	Adsorption energy	Rigid adsorption energy	Deformation energy	dE_ad_/dN_i_
-274.536	-291.6	-334.124	42.52391	-291.6

### Adsorption studies

Adsorption behavior further explains the corrosion inhibition process. The data was analyzed using a variety of adsorption isotherms, including Langmuir, Temkin, and Freundlich. Tables 1 and 2 show the expected surface coverage and inhibitory efficiency based on PDP and EIS measurements.

#### Langmuir adsorption isotherm

Surface coverage (θ) was estimated using the PDP and EIS methods and a suitable adsorption isotherm fitting to analyze inhibitor adsorption. The Langmuir adsorption isotherm provided the best relationship, as shown in Eq. 6.

6 $$\:\frac{\text{C}}{{\uptheta\:}}\:\:\:=\:\:1/{\text{K}}_{\text{a}\text{d}\text{s}}\:+\:\text{C}$$

**Fig. 13:**
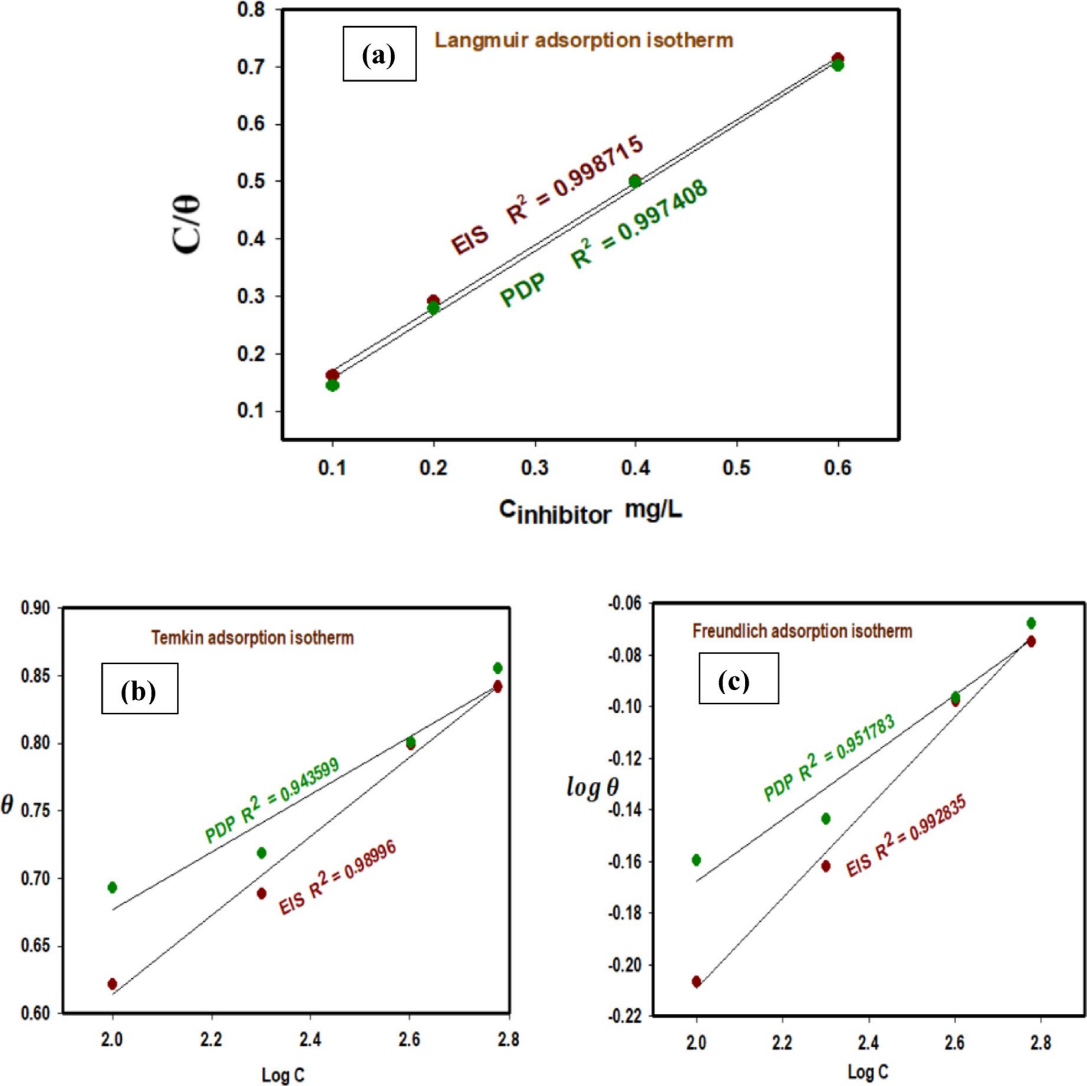
Adsorption isotherm plots for the adsorption of Cu_2_L_2_ nanocomplex on the SS surface from the binary acid mixture. (**a**) Langmuir, (**b**) Temkin, and (**c**) Frendlish isotherm.

*K*_ads_ is the equilibrium constant, and C is the inhibitor concentration. The plots of C/θ vs. *C* are straight lines with almost unit slopes, as given in Fig. 13a. It is found that the regression coefficients are very close to one (correlation coefficients, R^2^ of 0.997408 and 0.998715 were obtained for the PDP cand EIS methods, respectively), which indicates that adsorption of Cu_2_L_2_ nanocomplex on the 316 L stainless steel surface obeys Langmuir adsorption isotherm. The Langmuir adsorption isotherm assumes that all adsorption sites are equivalent and that the binding of particles occurs independently of nearby occupied sites^64^. The value of *K*_ads_ calculated from the isotherm fit in Fig. 13a was obtained as 1.3765 ppm^− 1^ and 1.5077 ppm^− 1^ for polarization and EIS methods, respectively. The inhibition efficiency of a compound depends on many factors, including the electronic structure, number of adsorption centers, mode of interactions with the metal surface, molecular size, and chemical properties of the inhibitor being adsorbed. It is well known that metal has an affinity toward nitrogen-, sulfur-, and oxygen-bearing ligands^65^. The electrochemical experiments reveal that stainless steel corrosion is retarded in the presence of Cu_2_L_2_ nanocomplex. The inhibitor molecules block the surface of stainless steel via an adsorption mechanism. The macromolecules of the Cu_2_L_2_ nanocomplex are electrostatically adsorbed on the 316 L stainless steel surface by donor-acceptor interactions between the π-electrons of fused benzene rings and the vacant d-orbitals of iron atoms. According to the polarization results, Cu_2_L_2_ nanocomplex follows the mixed inhibition mechanism. Some of the organic molecules in the Cu_2_L_2_ nanocomplex may get protonated, and these cationic forms may adsorb directly on the cathodic sites of the 316 L stainless steel and reduce the hydrogen evolution reaction. On the other hand, Cu_2_L_2_ nanocomplex may adsorb on the steel anodic sites through the π-electrons of the aromatic rings and the lone pair of electrons of heteroatoms, thereby inhibiting the anodic dissolution of 316 L stainless steel. Hence, by following the above mechanism, Cu_2_L_2_ nanocomplex shows the mixed inhibition behavior on the 316 L stainless steel surface in the binary acid mixture solution.

#### Emkin adsorption isotherm

Equation 7 states that the extent of surface coverage is proportional to the inhibitor concentration and the adsorption equilibrium constant *K*_ads_.

7 $$\:\:\text{e}\text{x}\text{p}(-2a\:\theta\:)\:=\:{K}_{ads}\:\times\:\:C$$

where a is an appealing parameter and *K*_ads_ is the adsorption constant. Linear graphs in Fig. 13b confirm that the adsorption follows the Temkin adsorption isotherm. Table 5 contains the adsorption parameters estimated from this figure.

#### Freundlich adsorption isotherm

The Freundlich isotherm (Eq. 8) shows a relationship between *θ* and inhibitor concentration C.

8 $$\:\text{l}\text{o}\text{g}\:\theta\:\:=\:\text{l}\text{o}\text{g}\:{K}_{\text{a}\text{d}\text{s}}\:+\:n\text{l}\text{o}\text{g}\:C$$

where n is an empirical constant, and the other symbols have the same meaning. Figure 13c illustrates a linear relationship between log *θ* and log *C*, with slope *n* and intercept log *K*_ads_. Adsorption investigated utilizing Langmuir, Freundlich, and Temkin isotherms revealed that experimental results met each of the three adsorption isotherms with high linearity. The best-fit isotherm’s criteria are based on the higher correlation coefficient *R*^2^. A higher *K*_ads_ value implies that the inhibitor is well-adsorbed on the SS surface. According to Table 5; Fig. 13a, the fitting was obtained using the Langmuir adsorption isotherm with high correlation coefficients (*R*^2^) compared to those for the other two models, Temkin and Freundlich isotherms. Table 5 shows that Δ*G*_ads_ values were negative, indicating that inhibitor adsorption onto the SS surface occurred spontaneously through physiosorption. Many researchers use adsorption isotherms to investigate the adsorption mechanism^33^.

Usually, $\:{{\Delta\:}\text{G}}_{ads}^{o}$ is computed from *K*_ads_ using Eq. 9:

9 $$\:{\text{K}}_{\text{a}\text{d}\text{s}}\:=\:1/55.5\:\text{e}\text{x}\text{p}\:(-({{\Delta\:}\text{G}}_{ads}^{o}/\text{R}\text{T})$$

$\:{{\Delta\:}\text{G}}_{ads}^{o}\:$is the standard free energy of inhibitor adsorption, 55.5 is the molar concentration of water in the solution, R is the gas constant, and T is the absolute temperature. Table 5 shows the calculated $\:{{\Delta\:}\text{G}}_{ads}^{o}$ for the Cu_2_L_2_ inhibitor at 298 K using the previously mentioned equation. The electrostatic interaction between the charged inhibitor molecules and the charged electrode (physiosorption) results in $\:{{\Delta\:}\text{G}}_{ads}^{o}$ values of -20 kJ mol^− 1^. More negative values than − 40 kJ mol^− 1^ indicate charge transfer from the inhibitor to the metal surface, resulting in a coordination bond (chemisorption)^65^. The observed adsorption is owing to the inhibitor’s inclusion of a broader range of chemical substances, some of which can be chemically adsorbed, while others can be physically adsorbed. The negative values of $\:{{\Delta\:}\text{G}}_{ads}^{o}$ (less than − 20 kJ mol^− 1^) obtained in this work suggest that the inhibitor adsorption process on the SS sample in a binary mixed acidic solution is spontaneous and follows the physiosorption approach.

**Table 5:** Adsorption parameters for the adsorption of Cu_2_L_2_ nanocomplex on the SS surface.

Isotherm	Technique	Slope	K_ads_ ppm^− 1^	$\:{{\Delta\:}\text{G}}_{ads}^{o}$ kJ mol^− 1^
Langmuir	PDP	1.105188	1.3765	-10.7425
	EIS	1.092482	1.5077	-5.2408
Temkin	PDP	0.212948	0.0721	-3.4356
	EIS	0.292396	0.9902	-9.9264
Freundlich	PDP	0.120551	0.3886	-7.6090
	EIS	0.174957	0.2528	-6.5438

### Effect of temperature on the Inhibition efficiency

**Fig. 14:**
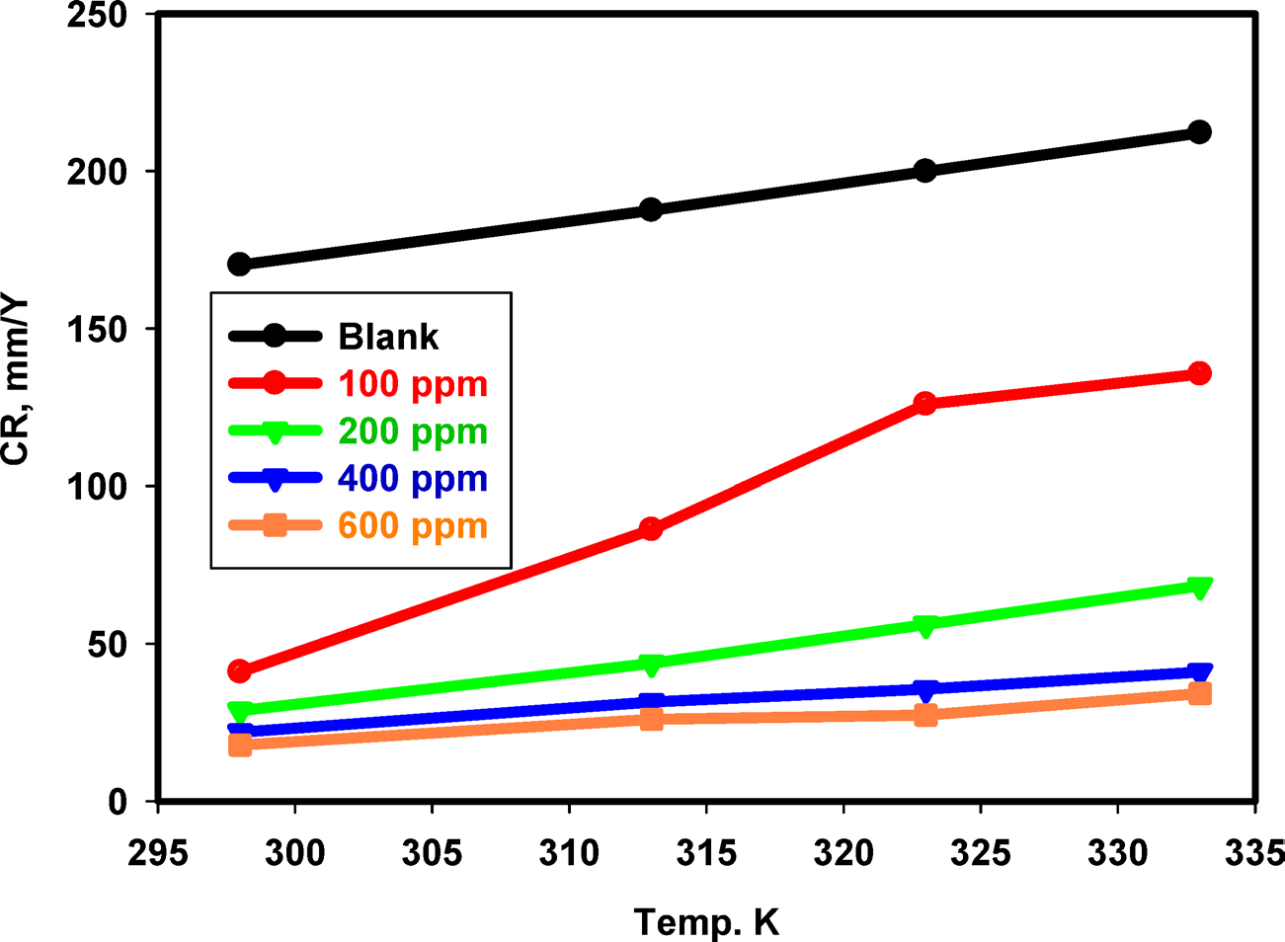
Effect of temperature on the corrosion rate of 316 L SS in the binary acid mixture without and with a different Cu_2_L_2_ nanocomplex.

The corrosion rates of 316 L SS as a function of temperature in the binary acid mixture without and with different Cu_2_L_2_ nanocomplex concentrations are shown in Fig. 14 using weight loss measurements. At all studied temperatures, inhibitor addition slightly reduced the corrosion rate when compared with the blank solution. To compute activation thermodynamic boundaries of the corrosion process, Arrhenius Eq. 10 and transition state theory Eq. 11 were utilized^65^.

10 $$\:{C}_{R}=A\:exp\left(\frac{{E}_{a}}{RT}\right)$$

11 $$\:CR=\frac{RT}{Nh}exp\frac{{\varDelta\:S}_{a}}{R}exp\left(-\frac{{\varDelta\:H}_{a}}{RT}\right)$$

**Fig. 15:**
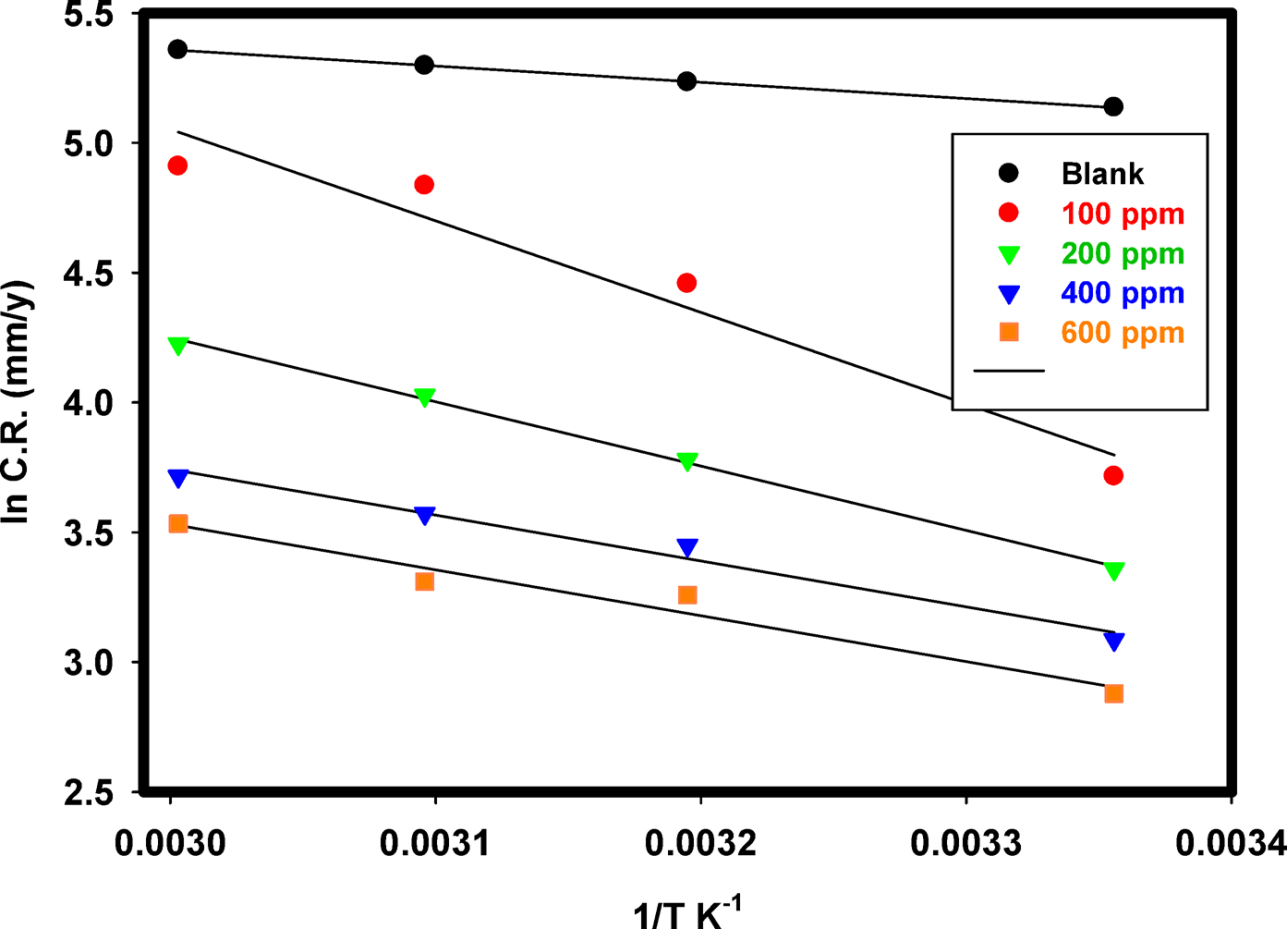
A plot of ln *CR* vs. (1/*T*) for 316 L SS in the binary acid mixture without and with a different Cu_2_L_2_ nanocomplex.

**Fig. 16:**
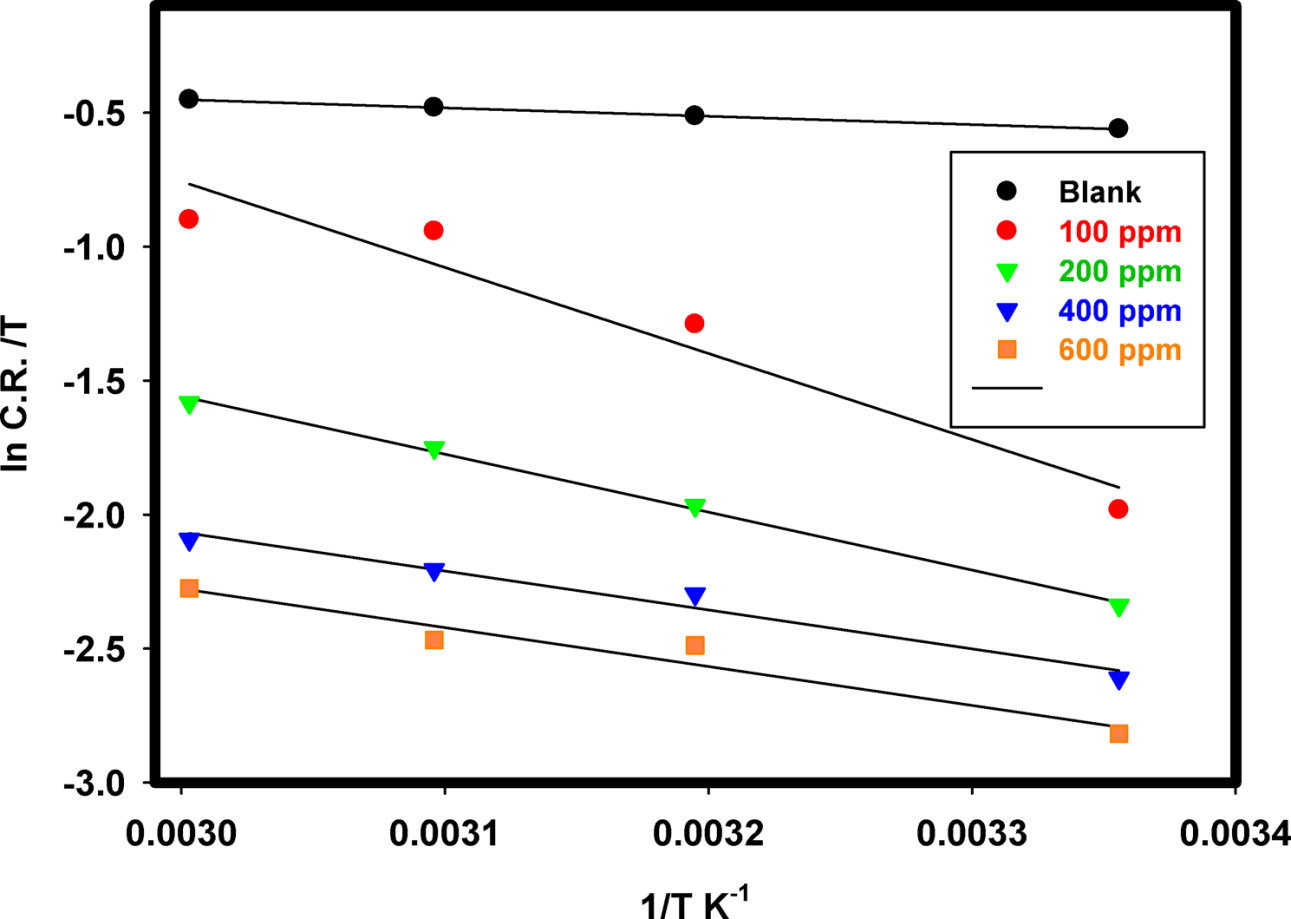
A plot of ln (*CR*/*T*) vs. (1/*T*) for 316 L SS in the binary acid mixture without and with a different Cu_2_L_2_ nanocomplex.

Where *E*_a_ is the activation energy, *R* is the gas constant, *A* is the pre-exponential factor of Arrhenius, *h* is the Planck constant, *N* is Avogadro’s number, Δ*S*_a_ is the entropy, and Δ*H*_a_ is the enthalpy of activation. Figure 15 depicts the Arrhenius curve of ln(CR) vs. (1/T) for 316 L SS in a binary acid mixture without and with different Cu_2_L_2_ nanocomplexes. The slope of the linear plot is -*E*a/*R*. The activation energy was determined without and with the studied inhibitor and found to be 5.2032 kJ mol^− 1^ for the blank and 14.681 kJ mol^− 1^ in the presence of 600 ppm Cu_2_L_2_ nanocomplex. It has been observed that the inhibited solution has a greater *E*_a_ value than the uninhibited solution, owing to the creation of an adsorbed film on the surface with increasing thickness, dramatically reducing SS dissolution. The linearized transition-state theory yielded the apparent activation enthalpy ∆*H*_*a*_ and entropy ∆*S*_a_ values, as presented in Table 6. Figure 16 shows plots of ln (*CR*/*T*) versus 1/*T* for 316 L SS in a binary acid mixture without and with different Cu_2_L_2_ nanocomplexes. The linear relationship has a slope of (-∆*H*_a_/*R*) and an intercept of (ln (*R*/*Nh*)+∆*S*_a_/*R*), where the enthalpy and entropy were determined, respectively. The enthalpies are 2.5893 and 12.0680 kJ mol^− 1^ for the blank and inhibited solutions. Positive ∆*H*_a_ values suggest an endothermic activation corrosion process, resulting in the gradual breakdown of SS. The entropy in the absence and presence of 600 ppm inhibitor is -193.5 and − 189.2 kJ mol^− 1^. Negative values of ∆*S*_a_ indicate a decrease in the disorder throughout the transition from reactants to activated complexes with metal.

**Table 6:** Thermodynamic activation parameters obtained by the weight-loss method.

Conc. ppm	A	*R* ^2^	E_a_ kJ mol^− 1^	∆H_a_ kJ mol^− 1^	∆S_a_ kJ mol^− 1^
**Blank**	1.9	0.99971	5.2032	2.5893	-193.5
**100**	2.7	0.94668	29.314	26.7007	-123.7
**200**	2.4	0.99816	20.591	17.9781	-156.5
**400**	2.2	0.98210	14.698	12.0847	-178.4
**600**	2.1	0.96190	14.681	12.0680	-189.2

### SEM and EDX spectra

**Fig. 17:**
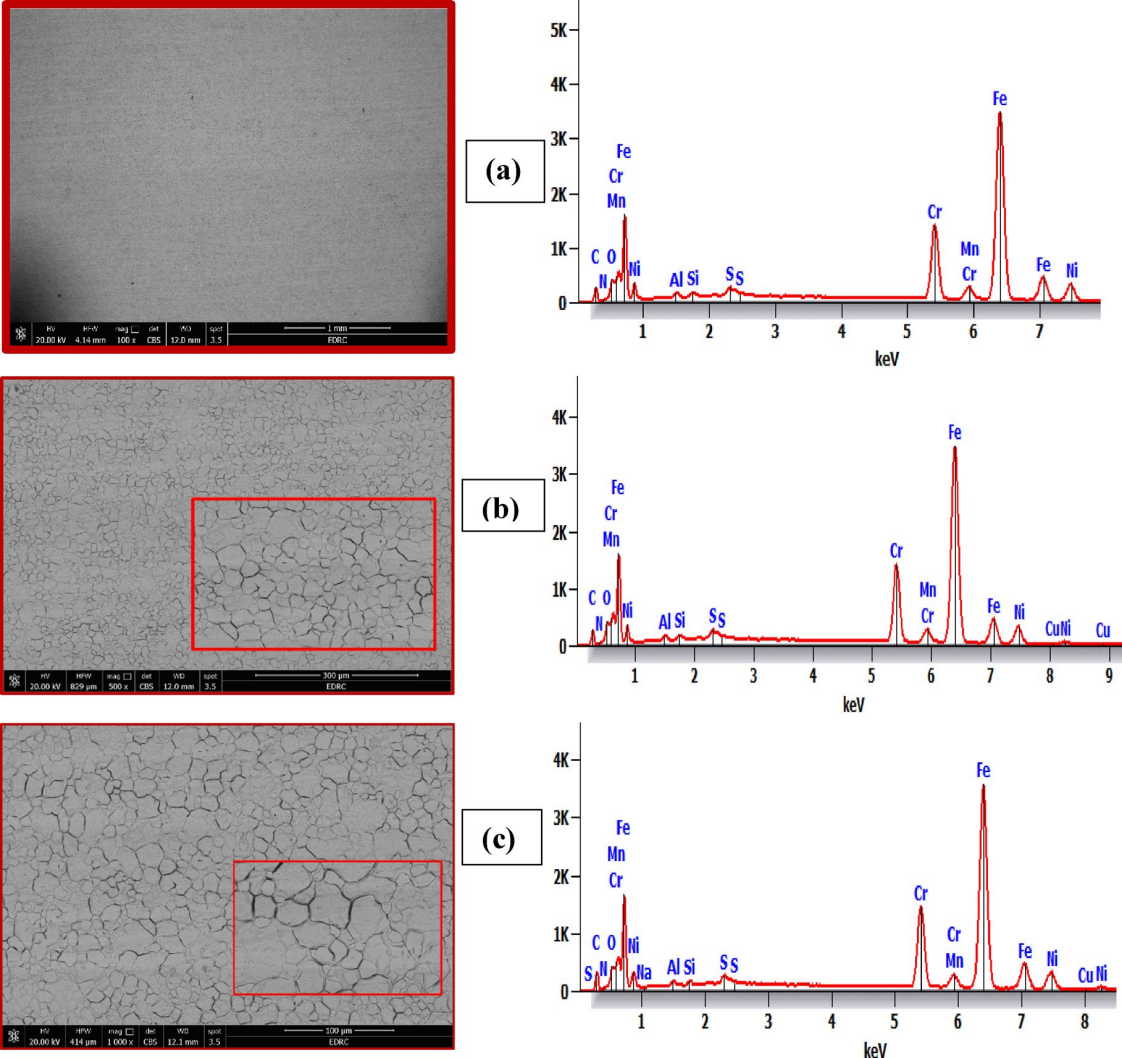
SEM images and EDX spectra for the 316 L SS sample in the binary acid mixture with Cu_2_L_2_ nanocomplex: (a) + 600 ppm, (b) + 100 ppm, and (c) the blank.

To further explore the corrosion inhibition of Cu_2_L_2_ nanocomplex, the SEM micrographs are obtained for 316 L stainless steel samples that were immersed in the binary acid mixture containing different concentrations (600 and 100 ppm) from Cu_2_L_2_ nanocomplex and in the blank (Fig. 17). In this figure, (a) and (b) presents stainless steel sample immersed in the acid mixture + 600 ppm and + 100 ppm Cu_2_L_2_ nanocomplex, respectively; and (c) presents stainless steel sample immersed in the binary acid mixture alone. As shown in Fig. 17(c), the stainless-steel surface was highly compromised without the nanocomplex, where the surface was rough and porous. On the other hand, in Figs. 17(a) and (b), the SS surfaces become less deteriorated and coherent, especially in the presence of 600 ppm nanocomplex, relevant to the surface immersed in the blank. The damage to the metal surface has diminished in the presence of the nanocomplex, which is ascribed to the formation of a protective layer by the constituents of the nanocomplex. This observation indicates that nanocomplex hinders the dissolution of iron and thereby reduces the corrosion rate of 316 L stainless steel in the binary acidic mixture. It is well known that the first step in inhibiting acid corrosion is the adsorption of inhibitor molecules onto the metal surface. Due to the electrostatic attraction, the cationic Cu_2_L_2_ nanocomplex molecules are physically adsorbed, and a high inhibition effect is expected. According to the EDX data, the percentage of elements on 316 L SS in the binary mixture in the presence and absence of Cu_2_L_2_ nanocomplex is depicted in Table 7. The EDX spectrum of the blank sample indicates peaks representing the five main elements (Fe, Cr, Ni, Mn, Si, and C) of 316 L SS. Peaks attributable to copper (Cu) could be components from the Cu_2_L_2_ ligand that should show up with the Cu_2_L_2_ nanocomplex. This nanocomplex may cause the percentages of Fe and Ni to drop while the rate of copper should rise.

Cu₂L₂ protection promotes the retention of passive components, as confirmed by Cr/Ni enrichment. Base metal dissolution is reduced because of corrosion inhibition. Cu₂L₂ stabilizes Cr₂O₃ by reducing defect density, forming a protective layer to deter acid penetration. Cu₂L₂ combines with Ni²⁺, generating an insoluble Ni-inhibitor layer. Cu₂L₂ limits Fe loss by blocking the active anodic sites. Adsorbed Cu₂L₂ binds to Cr₂O₃ and fills flaws in the passive films. Cu₂L₂ maintains these elements at 600 ppm, allowing EDX to detect them. EDX reveals Cr and Ni enrichment on 316 L SS with Cu₂L₂, which supports efficient corrosion inhibition. Cu₂L₂ adsorption inhibits Fe dissolution, resulting in the accumulation of passive elements (Cr/Ni). Ni forms a protective coating with the inhibitor, which improves re-passivation. Cu₂L₂ plugs flaws in the Cr₂O₃ layer, decreasing acid permeability. Thus, elemental redistribution confirms Cu₂L₂’s function in passive film stabilization.

**Table 7:** The percentage of elements present on 316 L SS in the binary acid mixture with different concentrations from Cu_2_L_2_ nanocomplex and in the blank.

Code	Media	C	*N*	O	Al	Si	S	Cr	Mn	Fe	Ni	Cu
**a**	**600 ppm**	2.3	0.7	1.1	0.3	0.3	0.8	15.8	1.5	68.3	10.1	0.5
**b**	**100 ppm**	2.2	0.6	1.0	0.3	0.3	0.8	15.7	1.5	68.6	8.6	0.3
**c**	**Blank**	2.1	--	0.9	0.25	0.29	--	15.4	1.3	68.7	8.5	0.2

### Inhibition mechanism of Cu₂L₂ nanocomplex

The inhibition mechanism involves synergistic interactions at the SS/solution interface, elucidated through combined electrochemical/surface analyses. 316 L SS corrosion involves simultaneous anodic dissolution and cathodic reduction reactions:

Anodic reactions.

Fe → Fe^2+^ +2e^−^ (Dominant).

Cr → Cr^3+^ + 3e^−^, Ni → Ni^2+^ +2e^−^ (Alloy components).

Cathodic reactions.

2 H^+^ + 2e^−^ → H_2_ (H^+^ reduction in HCl).

NO_3_^−^ +2 H^+^ +2e^−^ → NO_2_ + H_2_O (NO_3_^−^ reduction in HNO_3_).

Cl⁻ ions penetrate the oxide layer, allowing for localized corrosion. HNO₃’s oxidizing tendency promotes dissolution but may cause passivation at high potentials. Increased corrosion rate is due to aggressive attack by Cl⁻ and NO₃⁻ ions and destruction of the passive film (Cr₂O₃).

Inhibitor adsorption on the active sites (Fe/Cr/Ni) blocks dissolution:

Fe + Cu_2_L_2_ → Fe — Cu_2_L_2_.

**Fig. 18:**
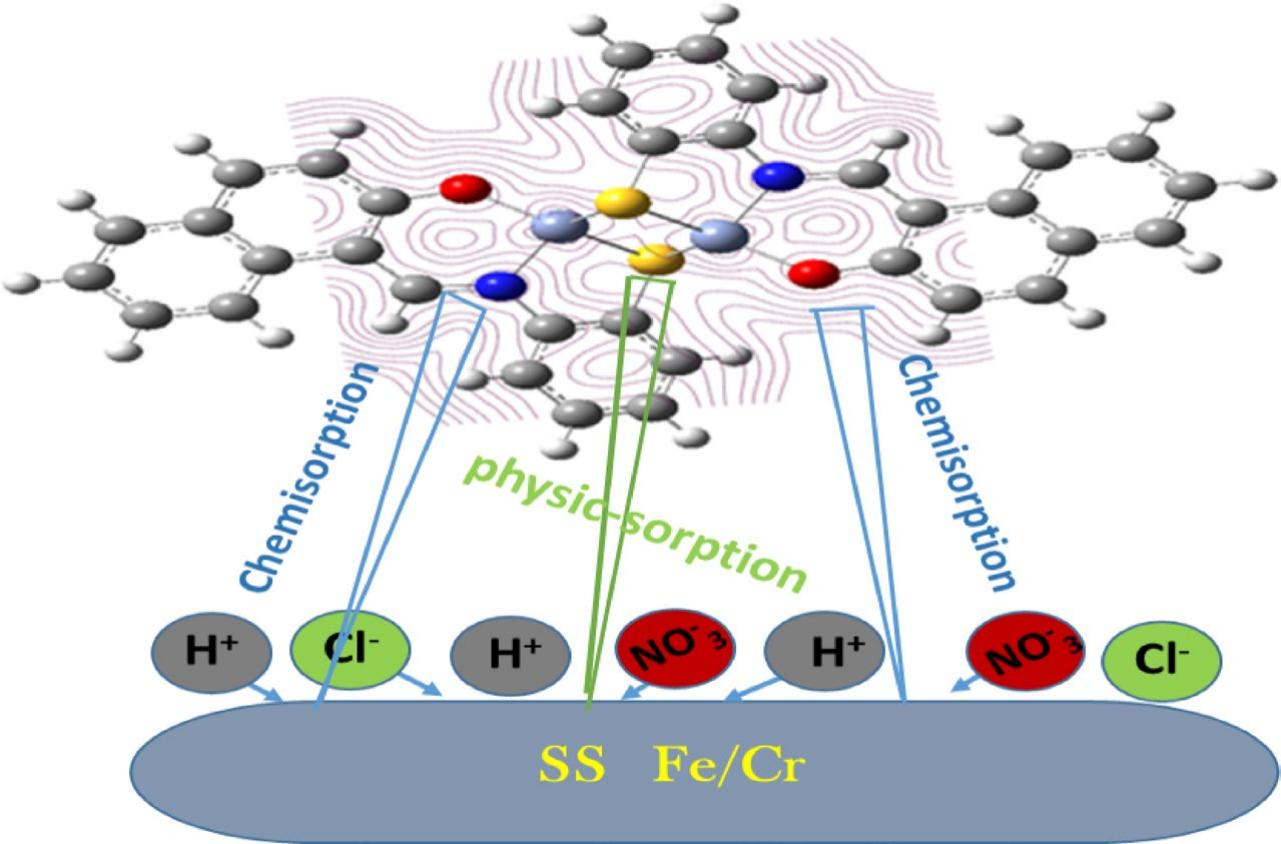
Inhibition mechanism of the Cu_2_L_2_ nanocomplex on stainless steel.

Stainless steel corrodes faster in HNO₃ + HCl due to the combination of Cl⁻ ions attack and NO₃⁻ ions oxidation. Nanocomplex Cu₂L₂ preferentially adsorbs on anodic sites by sharing electrons with Fe atoms. Coordinate bonding between electron-rich heteroatoms (N, O) in the ligand and vacant d-orbitals of Fe/Cr atoms (dominant). Electrostatic attraction between protonated ligands and negatively charged surface sites (via adsorbed Cl⁻). Stabilization of passive elements (Cr/Ni) through ligand-backbonding, enhancing oxide layer cohesion. ΔG°_ads_ = − 10.7425 kJ/mol confirms spontaneous physico-sorption (calculated from Langmuir isotherm). Lateral ligand-ligand interactions enhance film cohesion at high concentrations. Central Cu ions cross-link ligands into polymeric networks, resisting acid erosion. Inhibitor alters the Fe dissolution pathway via surface complexation. A mechanistic illustration (Fig. 18) depicts how Cu₂L₂ forms a hydrophobic, cross-linked film stabilizing passive elements (Cr/Ni) and blocking aggressive ions. A reducing acid that promotes active metal dissolution and localized pitting via chloride ion adsorption. A strong oxidizer HNO₃ induces passivation but can also cause dissolution at high concentrations. Cu₂L₂ forms an adhesive monolayer on the steel surface. This layer works as a barrier, preventing hostile ions like H⁺, Cl⁻, and NO₃⁻ from accessing the metal surface. Cu₂L₂ may help restore the passive film by forming a mixed oxide-hydroxide layer with Cu, Fe, and Cr. This improves surface resistance, reduces corrosion current, and positively shifts the corrosion potential.

## Conclusions

Cu_2_L_2_ nanocomplex was synthesized and tested as a green corrosion inhibitor for 316 L stainless steel samples in a harsh binary acid solution (1 M HCl + 1 M HNO_3_) using weight loss (WL), open circuit potential (OCP), potentiodynamic polarization (PDP), electrochemical impedance spectroscopy (EIS), surface morphology analysis, and theoretical quantum methods such as density functional theory (DFT) and molecular dynamic simulations (MD). The effects of factors such as concentration, exposure time, and reaction temperature on the inhibition efficiency of the nanocomplex were investigated. Generally, the corrosion rate of stainless steel decreases with increasing Cu_2_L_2_ dose in the acidic medium or prolonged immersion time and increases with raising the temperature. According to PDP tests, adding the inhibitor in concentrations ranging from 100 to 600 ppm mainly decreases the cathodic and anodic currents with a slight shift in *E*_corr_ towards more positive values, indicating that the Cu_2_L_2_ nanocomplex acts as a mixed-type inhibitor with a predominant anodic trait. At 600 ppm, the inhibition efficiency achieves an excellent value of 85.56%. Following the EIS data, the charge transfer resistance rises continuously, and the double-layer capacitance decreases with increasing Cu_2_L_2_ nanocomplex concentration. Values for the % inhibitory efficiency derived from PDP and EIS measurements are almost similar. The adsorption isotherm was interpreted as the adsorption of Cu_2_L_2_ nanocomplex over the SS surface, with an R^2^ value of 0.998715 (equivalent to 1), implying that the inhibitor molecules obscured a classic adsorption region when deposited on the metal/solution interface, and significantly reduced corrosion caused by the aggressive acidic environment. The active molecules from the Cu_2_L_2_ nanocomplex enhance the material performance under such harsh conditions and inhibit corrosion on the specimen surface, as confirmed by the SEM images and EDX analysis. These results suggest that Cu_2_L_2_ nanocomplex may be a sustainable substitute for the current generation of synthetic corrosion inhibitors.

Furthermore, computational DFT and MD simulation experiments demonstrated that the Cu_2_L_2_ inhibitor exhibits flat orientation on the SS surface, creating a stronger and more protective layer. The combination of empirical and computational methodologies revealed essential details about the mechanism underlying the Cu_2_L_2_ nanocomplex’s inhibitory performance on the SS surface. These findings pave the way for future studies, applications, and guidance in green corrosion inhibitors.

## Supplementary Information

Below is the link to the electronic supplementary material.

Supplementary Material 1

## Data Availability

All data generated or analyzed during this study are included in this article and available from the corresponding author, F.E.-T.H., upon reasonable request.
