# Supplementary material for: Synthesis of copper schiff base nanocomplex as green inhibitor for 316 L stainless steel corrosion in an aggressive acidic environment
Source: Sci Rep. 2025 Oct 20;15:36486. doi: 10.1038/s41598-025-21933-3 (PMC12537853; doi:10.1038/s41598-025-21933-3)
Supplement: Supplementary file 1 — Supplementary Material 1 [file 41598_2025_21933_MOESM1_ESM.docx]

**Synthesis of** **copper Schiff base nanocomplex as green inhibitor for 316L stainless steel corrosion in an aggressive acidic environment**

**Ghalia A. Gaber****^1*^,** **Shimaa Hosny^2^, Fakiha El-Taib Heakal^3*^**

^1^ *Chemistry Department, Faculty of Science (Girls), Al-Azhar University, Nasr City, Cairo11754, Egypt.*

([ghaliaasaid@azhar.edu.eg](mailto:ghaliaasaid@azhar.edu.eg))

*^2^Chemistry Department, Faculty of Science, New Valley University, El-Kharga, 72511, Egypt*

^3^*Chemistry Department, Faculty of Science, Cairo University, Giza 12613, Egypt.* ([hfakiha@cu.edu.eg](mailto:hfakiha@cu.edu.eg))

Fig. S1. Open circuit potential (OCP) of the Cu₂L₂ nanocomplex at 400 ppm in acidic medium (HCl + HNO₃) recorded for 90 min, showing that a quasi-stable potential is already reached within the first 30 min.
